# Advances in Josephson Junction Materials and Processes Toward Practical Quantum Computing

**DOI:** 10.1002/advs.78427

**Published:** 2026-10-28

**Authors:** Hyunseong Kim, Gyunghyun Jang, Seungwon Jin, Jaewon An, Dongbin Shin, Hyeon‐Jin Shin, Jie Luo, Akel Hashim, Irfan Siddiqi, Yosep Kim, Long B. Nguyen, Hoon Hahn Yoon

**Affiliations:** ^1^ Applied Mathematics and Computational Research Division Lawrence Berkeley National Laboratory Berkeley California USA; ^2^ Department of Physics University of California Berkeley California USA; ^3^ Department of Semiconductor Engineering Gwangju Institute of Science and Technology (GIST) Gwangju Republic of Korea; ^4^ Department of Physics Korea University Seoul Republic of Korea; ^5^ Department of Physics and Photon Science Gwangju Institute of Science and Technology (GIST) Gwangju Republic of Korea; ^6^ The Max Planck Institute for the Structure and Dynamics of Matter (MPSD) and the Center for Free Electron Laser Science (CFEL) Hamburg Germany; ^7^ Anyon Computing Inc. Emeryville California USA

**Keywords:** emerging materials, Josephson junctions, nanofabrication, quantum computing

## Abstract

The Josephson junction (JJ) is the fundamental nonlinear building block of superconducting quantum technologies. Its macroscopic quantum tunneling physics underpins superconducting quantum computing, sensing, and communication, but scaling these platforms to utility‐scale architectures places increasingly stringent demands on junction materials, interfaces, and fabrication. Practical quantum computing requires high reproducibility, low power consumption, tunability, a small device footprint, and resilience to noise and defects. This review surveys how advances in materials science, device characterization, and nanofabrication are addressing these challenges and redefining the figures of merit for next‐generation JJs. This review also examines the evolution of fabrication strategies, from conventional multi‐angle evaporation to foundry‐compatible superconducting processes and the integration of emerging junction materials. Progress along these directions will determine how rapidly JJs move from laboratory‐scale components to the foundation of industrial‐scale quantum processors.

## Graphical abstract

Emerging materials for quantum computing, including crystalline materials, two‐dimensional van der Waals materials, two‐dimensional electron gas systems, ferromagnets, and unconventional (e.g., d‐wave) superconductors, offer opportunities to engineer next‐generation Josephson junctions. By leveraging material properties and interfacial design, these platforms enable low dissipation, noise protection, compact footprint, and in situ tunability, paving the way toward fault‐tolerant and scalable superconducting quantum processors.

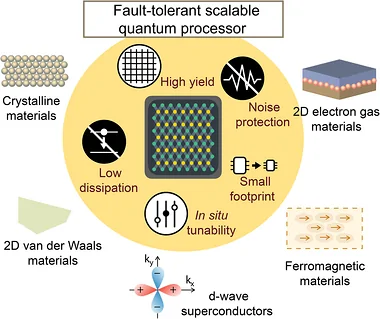

## 1 Introduction

From the outset of the Industrial Revolution, numerous technological breakthroughs have emerged through cross‐pollination among diverse scientific disciplines. A paradigmatic example is magnetic resonance imaging, which emerged from the convergence of nuclear physics, chemistry, medicine, mathematics, and computer science. In the modern era, the development of quantum technologies has also emerged from similar interrelationships among quantum physics, computer science, electrical engineering, and many other fields. Notably, the convergence of low‐temperature electronics, quantum optics, and materials science has given rise to circuit quantum electrodynamics [1], which governs the behavior of modern superconducting circuits. Horizontal progress across multiple fronts has propelled superconducting circuits as a leading solid‐state platform for scalable quantum technologies. These devices utilize the Josephson junction (JJ), which is the foundational building block of large‐scale quantum information processors [2, 3], the cornerstone of quantum sensing and metrological science [4], and the core unit for modular quantum networks. Moreover, JJ‐based devices have enabled the development of novel cryogenic microwave components, such as Josephson diodes [5], parametric amplifiers [6], and integrated circulators [7, 8, 9].

Since the first observation of the Josephson effect using ultra‐thin ${\mathrm{SnO}}_{x}$ barriers between Sn and Pb superconductors (SCs) [19], JJs have been implemented in a wide range of architectures, each instrumental in advancing transformative quantum technologies. Today, the exploration of novel junction materials continues to expand the quantum hardware landscape, as illustrated in Figure 1, while innovations in device design and characterization reveal complex phenomena observed in experiments. Concurrently, efforts to scale noisy intermediate‐scale quantum devices [20] have yielded valuable insights into the challenges and opportunities for building larger systems. The integration of advanced quantum characterization, validation, and verification tools with next‐generation Josephson circuits is poised to drive further innovation at the frontiers of quantum technology [21, 22]. For example, systematic monitoring of devices across varied geometries and carefully controlled environments has been key to identifying and mitigating material‐related energy losses in superconducting qubits [3, 23], enabling continuous improvements in scalability and performance.

**FIGURE 1:**
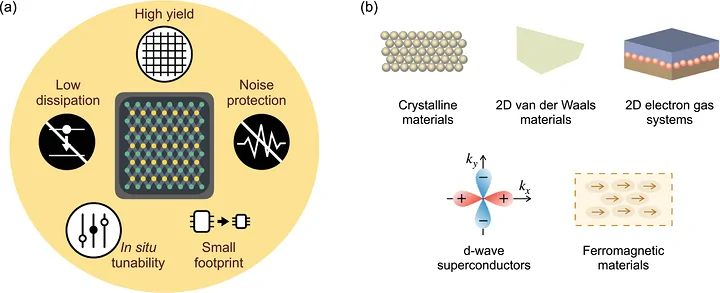
Promising material‐oriented strategies for practical superconducting quantum computing. (a) Current superconducting quantum processors suffer from various issues due to fabrication imperfections or system limitations, which can be resolved if qubits become low‐dissipative [10], noise‐protected [11], and frequency‐tunable [12]. Furthermore, higher individual‐qubit yield on a single processor [13] and smaller qubit size are highly beneficial for achieving a practical quantum computing era [14]. (b) The performance of superconducting qubits can be enhanced primarily by fabricating highly crystalline metals. Also, opportunities are seen in two‐dimensional van der Waals (2D vdW) materials [15], two‐dimensional electron gas (2DEG) systems [16], d‐wave superconductors [17], and ferromagnetic materials [18].

In this review, we examine ongoing efforts to advance JJs for practical quantum computing, focusing on the underlying physical principles and key performance and integration metrics, including reproducibility, robustness against noise and defects, in situ tunability, and device footprint. To address these figures of merit, we highlight opportunities enabled by novel junction materials and emphasize the importance of integrating advanced characterization techniques with innovative device architectures supported by creative fabrication methods. We also discuss the nanofabrication challenges inherent in developing next‐generation JJ‐based devices and the unique opportunities arising from overcoming these hurdles. Although the primary focus is quantum computing, the approaches described here are broadly applicable to other areas of quantum science and technology, including quantum sensing and quantum communication.

This review is structured as follows. First, Section 2 briefly revisits the Josephson effect, covering the fundamentals needed to understand the nonlinear behaviors of JJs. Section 3 surveys the key figures of merit crucial to the construction of superconducting quantum processors, encompassing tunnel barrier uniformity (3.1), energy loss mechanisms associated with the junctions (3.2), enhanced tunability (3.3), reduced device footprint (3.4), and cutting‐edge ideas about robust qubits derived from d‐wave SCs or JJs with a ferromagnetic insulating interlayer (3.5). Section 4 focuses on advanced nanofabrication techniques, highlighting large‐scale integration and process compatibility. Finally, Section 5 presents an outlook on the future of JJs in quantum processors, discussing emerging material platforms, scalable fabrication strategies, and insights from the historical evolution of the CMOS (complementary metal‐oxide‐semiconductor) integrated circuit (IC) chip industry. A list of abbreviations used in this review is provided in Appendix A.

## 2 Revisiting the Josephson Effect

In 1962, Brian Josephson predicted the dissipationless flow of a supercurrent between two superconducting electrodes separated by a thin insulating barrier, a phenomenon now known as the *Josephson effect* (Figure 2a) [24]. At the time, quantum tunneling was well established for normal (non‐superconducting) electrons; Josephson's key insight was that Cooper pairs could likewise tunnel coherently across a weak link. The subsequent experimental verification of this prediction [19, 25] led to Josephson being awarded the 1973 Nobel Prize in Physics. A particularly striking aspect of the Josephson effect is that the supercurrent depends on the relative phase difference between the superconducting order parameters, a concept that was both unexpected and conceptually profound at the time. In modern times, engineering the current‐phase relationship in Equation (1), its functional form, symmetry, and nonlinearity has become a central focus in developing novel JJs, driving fundamental research and technological advancements.

**FIGURE 2:**
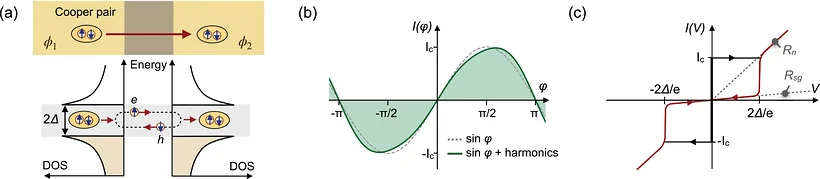
Transport mechanisms, current‐phase relation (CPR), and current‐voltage (I–V) characteristics in Josephson junctions (JJs). (a) Charge transport mechanisms in superconductor–insulator–superconductor (SIS) and superconductor–normal metal–superconductor (SNS) junctions. In SIS junctions, Cooper pairs tunnel coherently through the insulating barrier. In SNS junctions, phase‐coherent transport occurs via Andreev reflection, forming Andreev bound states (ABS) in the normal region that carry the supercurrent [26]. (b) CPR of JJs. SIS junctions in the tunneling limit exhibit a sinusoidal CPR, $I\left(\varphi \right)=I_{c}\sin\varphi$. In contrast, high‐transparency SNS junctions support ABS whose nonlinear phase dispersion yields a nonsinusoidal CPR containing higher harmonics [27]. (c) Nonlinear I‐V characteristics of JJs. The normal‐state resistance $R_{n}$ is defined from the slope at voltages above the superconducting gap, whereas the subgap resistance $R_{\mathrm{sg}}$ characterizes dissipative transport within the superconducting gap region [28].

The Josephson effect arises from the macroscopic quantum coherence of the superconducting condensate. Each SC is characterized by a macroscopic wavefunction $\psi =\sqrt{n}e^{i\phi }$, where n is the Cooper‐pair density and ϕ is the phase. The gradient of the phase governs the supercurrent flow, described by the probability current density $\vec{J}\propto Im\left[\psi^{*}\nabla \psi \right]$. For a uniform Cooper‐pair density, this simplifies to $\vec{J}\propto \nabla \phi$, showing that the supercurrent is proportional to the phase gradient. Therefore, when two SCs are weakly coupled through a tunneling barrier, the phase difference δϕ drives a dissipationless supercurrent. Due to the single‐valuedness of the condensate wave function, this current is a periodic function of δϕ with a period equal to 2π. Furthermore, time‐reversal symmetry (TRS) ensures that the current is an odd function of δϕ. In the weakly coupled tunnel junction regime, we obtain a characteristic sinusoidal current‐phase relation (CPR),

(1) $$I=I_{c}\sin\left(\varphi \right),$$

where $I_{c}$ is the critical current and φ≡δϕ.

In addition to the CPR, the Josephson effect also includes a dynamical component that links the time derivative of the phase difference to the junction voltage. This is commonly expressed as the second Josephson relation,

(2) $$\frac{d\varphi }{dt}=\frac{2\pi }{\Phi_{0}}V,$$

which follows directly from the Schrödinger equation and Faraday's law of electromagnetic induction. The dynamic aspect of the Josephson effect establishes a direct connection between the quantum phase and observable electrical quantities, serving as the foundation for applications such as voltage standards. Furthermore, Equation (2) directly leads to the Josephson energy‐phase relation,

(3) $$\begin{array}{cc} E(\varphi ) & =\int I\left(\varphi \right)Vdt=\int I_{c}\sin\left(\varphi \right)\frac{\Phi_{0}}{2\pi }d\varphi \\ & =-\frac{\Phi_{0}I_{c}}{2\pi }\cos\varphi =-E_{J}\cos\varphi , \end{array}$$

where $E_{J}$ is the so‐called *Josephson energy*. Equation (3) is fundamental to understanding and engineering systems in circuit quantum electrodynamics. It highlights the JJ's coherent and nonlinear properties, which enable a broad spectrum of quantum technologies.

While Equation (3) captures the fundamental sinusoidal dependence of the Josephson energy on the superconducting phase difference, it reflects an idealized tunneling limit in which only the first harmonic is significant. In general, in normal materials such as insulators, normal metals, and semiconductors (SMs), the CPR can exhibit substantially higher‐order Josephson harmonics, depending on the microscopic properties of the junction. According to microscopic theory, the dissipationless Josephson current arises from the lower‐level *Andreev bound state* (ABS), which forms due to multiple Andreev reflections at the interfaces between the SCs and the normal barrier, as shown in Figure 2a [29, 30]. In an Andreev reflection, an electron incident on the interface is retroreflected as a hole, effectively transferring a Cooper pair across the interface. In the short junction regime, repeated reflections that constructively interfere within the junction give rise to phase‐dependent bound states with energies given by Equation (4)

(4) $$E_{\mathrm{ABS}}\left(\varphi \right)=\pm \Delta \sqrt{1-\tau \sin^{2}\left(\varphi /2\right)},$$

where τ is the conduction channel's transmission probability, or its so‐called *transparency*, and Δ is the superconducting gap [31]. In the low temperature limit, the Josephson current is carried by the lower (negative) energy ABS. Summing over all conduction channels and applying the second Josephson relation, we arrive at the generalized CPR,

(5) $$\begin{array}{cc} I(\varphi ) & =\sum_{i}\frac{e\Delta }{\hbar }\times \frac{\tau_{i}\sin\left(\varphi \right)}{2\sqrt{1-\tau_{i}\sin^{2}\left(\varphi /2\right)}}\\ & =\sum_{i}\sum_{k}I_{ki}\sin\left(k\varphi \right), \end{array}$$

where the harmonic decomposition comes from the binomial series expansion (Appendix B). Similar to how the momentum operator generates translations through the quantum‐mechanical conjugacy between position and momentum, the conjugacy between charge and flux (phase) makes $e^{i\varphi }$ the translation operator for the Cooper‐pair number. Consequently, in the charge basis, the higher Josephson harmonics (k>1) correspond to effective multi‐Cooper‐pair tunneling processes [32]. In the low transparency limit, $\tau_{i}\ll 1$, the CPR reduces to the sinusoidal form of Equation (1). However, higher‐order harmonics become significant for highly transparent channels, indicating a strongly anharmonic CPR (Figure 2b). By tuning transparency through material choice, junction geometry, or electrostatic gating, one can sculpt the CPR to enhance qubit performance (see Section 3.5) [33].

While the CPR is naturally expressed in terms of the transmission probabilities $\tau_{i}$ of individual conduction channels, these microscopic quantities are not directly accessible experimentally. Instead, the Landauer formalism relates the junction's overall transparency to the normal‐state resistance $R_{n}$. The key properties of JJs must be accurately characterized before they can be integrated into a quantum application. A fundamental and practical method for this characterization is to extract the critical current $I_{c}$ from the normal‐state resistance $R_{n}$, measured right after the superconducting gap (Figure 2c). The resistance of a quantum conductor is related to the aggregate transparency of the conduction channels through Landauer's formula [34]:

(6) $$\frac{1}{R_{n}}=\sum_{i}\frac{e^{2}}{\pi \hbar }\tau_{i}.$$

Applying Equation (6) to the CPR in Equation (5), and considering the low‐transparency limit, leads to the well‐known Ambegaokar–Baratoff relation [35]:

(7) $$I_{c}=\frac{\pi \Delta }{2eR_{n}}.$$

This result directly estimates the JJ's critical current from normal‐state resistance measurements, enabling efficient yield characterization of superconducting quantum processors (Subsection 3.1). Importantly, it offers a noninvasive diagnostic tool that reduces the need for extensive cryogenic cycling, streamlining the fabrication and testing of large‐scale superconducting circuits. Notably, this relation is exact only in the tunneling limit, with deviations of up to 2× in the ballistic limit [32], providing a diagnostic of the transmission distribution.

Cryogenic characterization techniques provide essential insight into the transport properties of JJs under their actual operating conditions. One of the most informative tools is the current–voltage (I–V) measurement, which can be used to determine the critical current, the point at which a finite voltage develops across the junction, corresponding to the breakdown of the dissipationless supercurrent [36]. For tunnel junctions, if the junction exhibits leakage current, i.e., quasiparticle transport below the superconducting gap, a finite subgap voltage is characterized by the subgap resistance shown in Figure 2c. This resistance is inversely proportional to the population of thermally excited quasiparticles, which act as sources of decoherence in quantum devices [37]. Consequently, a considerable subgap resistance is crucial for quantum applications such as superconducting qubits (Section 3).

The detailed shape of the I–V contour offers additional information about the junction's internal dynamics [26, 38, 39, 40]. For example, hysteresis in the I–V curves typically signals an underdamped junction, where significant normal‐state resistance and junction capacitance contribute to inertial phase dynamics. Asymmetries that indicate a shifted or distorted CPR can be used to introduce nonreciprocal elements. Furthermore, when the junction is subjected to an oscillating voltage of frequency ω, the I–V curve exhibits a series of plateaus known as Shapiro steps, occurring at voltages V=νℏω/2e, where ν∈Z. In junctions exhibiting higher‐order Josephson harmonics, fractional Shapiro steps appear, indicating multi‐Cooper‐pair tunneling. As such, I–V characterization remains a cornerstone technique for probing JJs. Additional techniques, such as measuring the critical‐current modulation in response to an applied magnetic field or embedding a junction in a loop containing another well‐characterized junction, provide valuable insights into both spatial uniformity and the CPR [41, 42]. With a solid understanding of the JJ's transport properties, we now focus on its diverse quantum applications, which leverage its dissipationless transport and nonlinear behavior.

## 3 Quantum Computing

Superconducting circuits are among the most promising platforms for realizing practical quantum computers. Superconducting qubits leverage the well‐established principles of superconductivity and the Josephson effect to create quantum systems that can be controlled scalably [28]. Remarkable progress is evident in the growing qubit counts and increasingly complex quantum operations, enabling milestones such as quantum supremacy and quantum error correction [90, 91]. There is considerable effort to achieve a meaningful computational speedup over classical computing for practical applications, a critical benchmark often referred to as *quantum utility* [92]. In parallel with advances in quantum computing hardware, significant efforts are directed toward developing practical quantum algorithms suited for current and near‐term devices [93, 94, 95] and quantum simulations [96], with a particular emphasis on scientific applications [97, 98].

The core of superconducting qubit technology is currently the Al/${\mathrm{AlO}}_{x}$/Al tunnel junction. These junctions are the building blocks of contemporary qubits, providing nonlinear inductance that enables the manipulation of quantum states. Combining several junctions with a toolbox of superconducting capacitors and inductors enables the construction of various qubits with different properties [28, 99]. The transmon, as shown in Figure 3b, is the most common type of superconducting qubit due to its superior coherence properties and ease of fabrication. The qubit features a JJ shunted by a large capacitor, functioning as a nonlinear oscillator. Adding a second JJ in parallel forms a *superconducting quantum interference device* (SQUID), allowing the effective Josephson energy and hence the oscillator frequency to be tuned by the magnetic flux threading the loop.

**FIGURE 3:**
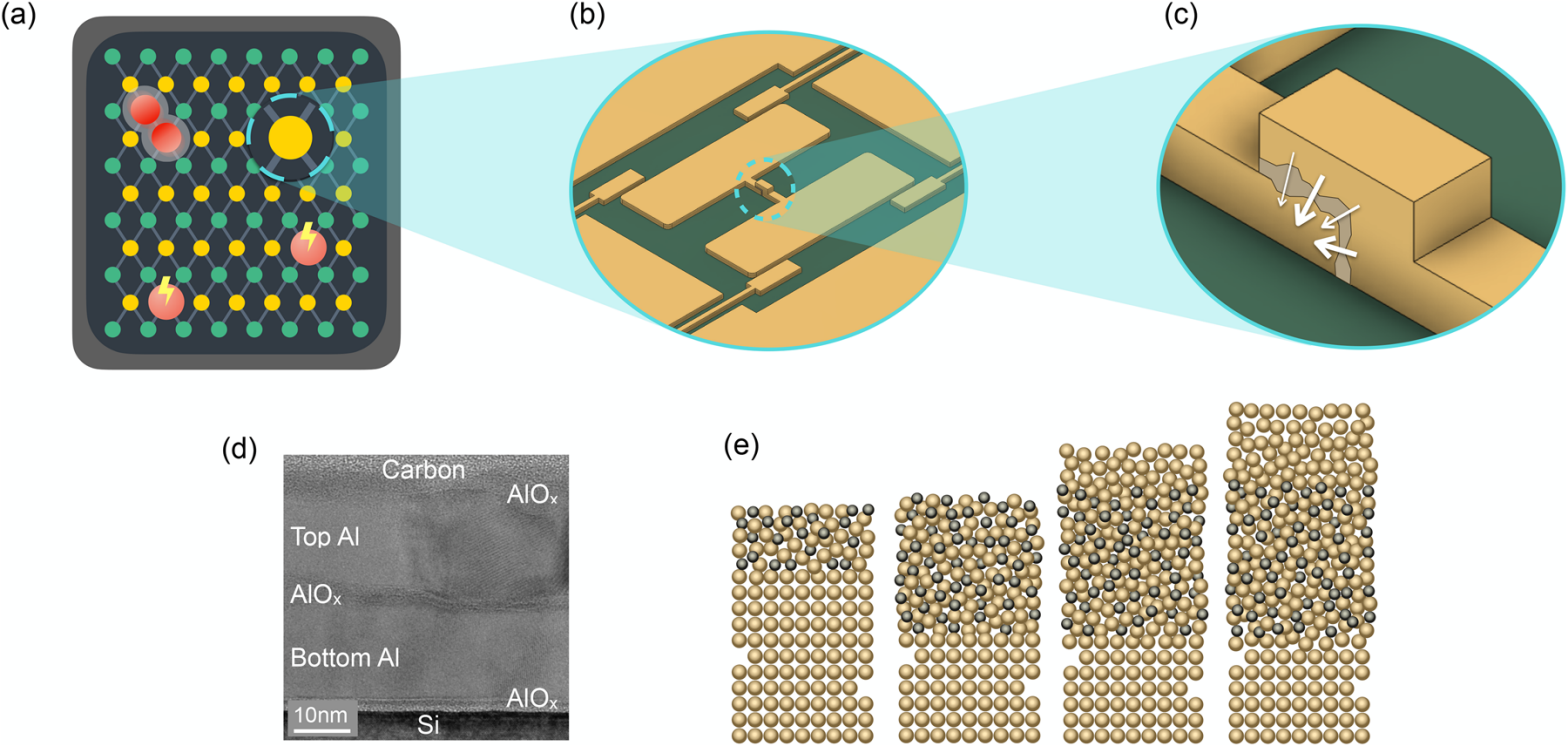
Josephson junction (JJ) imperfections. (a) Schematic of a superconducting quantum processor, highlighting defective outlier qubits caused by frequency collisions and defects due to interaction with the lossy environment. (b) A JJ connecting two large superconducting electrodes, forming a transmon qubit. Smaller structures adjacent to the pads enable capacitive coupling to external circuitry. These sizes are not to scale. (c) Illustration of spatial variations in supercurrent density (white arrows) arising from nonuniform junction thickness and surface roughness along the tunneling barrier [43, 44]. (d) Transmission electron microscopy (TEM) image of an Al/$\mathrm{Al}O_{x}$/Al junction fabricated on a silicon substrate. The few‐nanometer‐thick $\mathrm{Al}O_{x}$ layer with varying thickness largely determines the Josephson energy $E_{J}$. Reproduced with permission. [57] Copyright 2022, AIP Publishing. (e) Model of atomic structure evolution during Al/$\mathrm{Al}O_{x}$/Al junction formation, showing the oxidation and deposition process on a crystalline aluminum bottom electrode (yellow: aluminum, black: oxygen). Molecular dynamics simulations with the Streitz–Mintmire potential enable analysis of structural properties during junction formation under varying conditions, such as temperature, bond angle, and oxygen pressure [100].

The success of superconducting transmon qubits and qudits [101, 102, 103, 104, 105] has been driven by sustained efforts to improve coherence through horizontal approaches, such as reinforced shielding and filtering [106, 107, 108], material purification through advanced fabrication techniques [23], packaging [109], and optimization of qubit geometry [99, 110, 111, 112]. Interestingly, the transmon's simplicity makes it an ideal vehicle for probing the surrounding noisy environment and examining general decoherence mechanisms. Once perceived as an insurmountable obstacle, the strong coupling of superconducting qubits to their imperfect bath has proven valuable in exploring microscopic material defects that may affect other solid‐state platforms [113]. These newfound insights into noise properties have deepened our understanding of decoherence and play a pivotal role in developing new circuit platforms designed for superior noise resilience [114, 115].

As the number of qubits in quantum processors increases, solutions that work effectively at small scales may no longer suffice for large‐scale systems. Achieving high‐performance quantum computation at scale will require fundamentally new approaches. One critical challenge is that the device's worst‐performing qubit often constrains a quantum processor's performance [3, 91]. While significant progress has been made in improving average qubit performance, prioritizing uniformity across the entire device is now essential to ensure consistent reliability. Another pressing need is developing qubits with smaller footprints, as chip size imposes inherent physical limitations. Reducing the qubit size will enable higher qubit densities without compromising functionality. Moreover, implementing additional protection mechanisms could substantially improve qubit coherence times, further advancing device performance. Table 1 summarizes the key performance metrics, associated challenges, and opportunities for improvement. In the following discussion, we explore how innovative engineering methodologies for JJs could address these challenges and meet the demands of next‐generation superconducting quantum processors.

**TABLE 1:** Performance metrics for quantum computing and some relevant challenges for improvement.

Figures of Merit	Challenges	Opportunities
Reproducibility Yield Long‐term junction stability	Varying junction thickness [43, 44] Varying junction area [45, 46] Line‐edge roughness [47] Aging effect [48, 49]	Advanced fabrication with precise barrier thickness [50, 51, 52, 53] Novel junction materials [52, 53, 54, 55, 56] Post‐fabrication corrections [57, 58] Passivation and thermal annealing [48, 49, 59, 60]
Energy dissipation	Two‐level systems [61, 62] Quasiparticle tunneling [63, 64, 65]	Low‐loss crystalline tunnel barriers [53, 54, 55] Two‐dimensional van der Waals (2D vdW) materials and their heterostructures [15, 56, 66, 67, 68] Gap‐engineered electrodes [63, 64, 65, 69]
In situ tunability	Heat load and crosstalk due to flux tuning [70, 71] Footprint of flux loops	Gate‐tunable two‐dimensional electron gas (2DEG) systems [16, 72, 73] Gate‐tunable 2D vdW JJs [74, 75, 76, 77]
Device footprint	Large‐area circuit elements [78]	Merged‐element transmon qubits [79, 80] High‐κ 2D vdW tunnel barriers [81]
Noise‐protected encoding	Tuning Cooper‐pair parity [82, 83, 84]	Twisted d‐wave flakes [85, 86] Stacking d‐wave and s‐wave flakes [17] π‐junctions with ferromagnetic interlayers [87, 88, 89]

### 3.1 Yield and Reproducibility

Superconducting quantum processors, shown in Figure 3a, require the constituent qubits to be spectrally distributed to suppress spurious interactions and enable individual addressability [116]. This underscores the need to scale with exceptionally high JJ fabrication precision. While device yield may vary across different platforms [115], we explore here the transmon qubit as a representative example, as depicted in Figure 3b. Its computational transition frequency is given by

(8) $$\frac{\omega_{01}}{2\pi }\approx \frac{\sqrt{8E_{J}E_{C}}-E_{C}}{h},$$

where $E_{C}=e^{2}/2C$ is the charging energy set by the shunt capacitance C, and $E_{J}=\Phi_{0}I_{c}/2\pi$ is the Josephson energy determined by the critical current of the junction (see Equation 3). While capacitive elements with relatively large dimensions can be fabricated with high reproducibility, JJs are highly susceptible to fabrication‐induced variations. For instance, a 3% fluctuation in $I_{c}$ results in an approximately 1.5% change in $\omega_{01}$, corresponding to a 75 MHz shift for a transmon qubit designed to operate at 5 GHz, which is substantial relative to typical qubit frequency spacings of tens to a hundred megahertz [117].

Thus, high‐precision JJ fabrication is essential to maintain device yield in large‐scale superconducting quantum processors [13]. Although frequency‐tunable superconducting qubits can mitigate frequency collisions, precise $I_{c}$ control remains essential to maintain optimal qubit performance at noise‐resilient operating points [115]. Moreover, while post‐fabrication frequency‐tuning strategies can compensate for some fabrication‐induced variability [57, 58, 118], improving JJ reproducibility remains indispensable for scalable quantum processors.

The junction's critical current $I_{c}$ is primarily determined by its dimensions and the tunnel‐barrier material structure. Advances in shadow evaporation techniques, combined with optimized oxidation processes and bias corrections, have reduced variations in $I_{c}$ to within ∼2% across wafers [45, 46, 47, 119, 120]. However, conventional multi‐angle evaporation faces inherent challenges, including the mechanical instability of resist bridges, increased line‐edge roughness, and radial nonuniformities introduced by angled deposition, which may fundamentally limit achievable junction reproducibility. To overcome these limitations, researchers have developed fabrication approaches compatible with optical lithography and orthogonal dry etching [121, 122, 123, 124, 125], providing sub‐100‐nm critical dimension control and enabling scalable fabrication on 300‐mm wafers. Such etch‐based processes have achieved $I_{c}$ uniformity comparable to advanced shadow evaporation techniques [126]. A more detailed description of these fabrication techniques and their limitations is provided in Section 4.

Despite these advances, downstream effects inherent to junction physics, such as frequency shifts and parameter drifts, continue to limit the precision of frequency targeting. Even with tight control over the junction area and the thickness of the tunnel barrier, microscopic nonuniformities [43, 44], such as variations in local thickness in the junction or roughness in the electrode and barrier layers (Figure 3c,d), can induce substantial fluctuations in local critical current density, ultimately impacting junction consistency. Careful management of these microscopic effects, such as narrowing the transmission distribution, is therefore crucial for maintaining fabrication quality. Further improvements through oxidation optimization [50, 51] and epitaxial growth [52, 54, 55] allow for atomic‐scale control of tunnel barrier properties. As illustrated in Figure 3e, fabricating highly crystalline bottom electrodes promotes uniform oxidation and top‐electrode deposition [53, 100], improving junction uniformity. Furthermore, post‐fabrication treatments, such as thermal annealing [48, 49] and surface oxide passivation [59, 60] have been shown to mitigate frequency drift and enhance the long‐term stability of the device.

Beyond the frequency targeting achieved through process control, the intrinsic material quality of JJs plays a critical role in determining the scalability and yield of superconducting quantum processors. Localized defects near the junction, including two‐level systems (TLS) in tunnel barriers, quasiparticle generation from external radiation, and dielectric loss at material interfaces, introduce variability in qubit relaxation times and coherence properties (Figure 4, see Section 3.2). Tightening temporal variations in fidelity is crucial for fault‐tolerant quantum computation, as even a small number of low‐fidelity qubits can compromise quantum error‐correction performance [3, 22]. Addressing this challenge requires integrating efficient quality control strategies into fabrication processes. In particular, early detection of fabrication‐induced defects is essential for maintaining high device yield across large wafers. To this end, incorporating in situ optical defect inspection, a well‐established method in CMOS process flow, provides a promising route to identify and mitigate fabrication‐induced defects early in the process [127]. In conjunction with cryogenic measurement feedback, new junction processes may also benefit from high‐resolution material imaging techniques in Figure 5, such as transmission electron microscopy, scanning electron microscopy, and atomic force microscopy, when deployed at scale.

**FIGURE 4:**
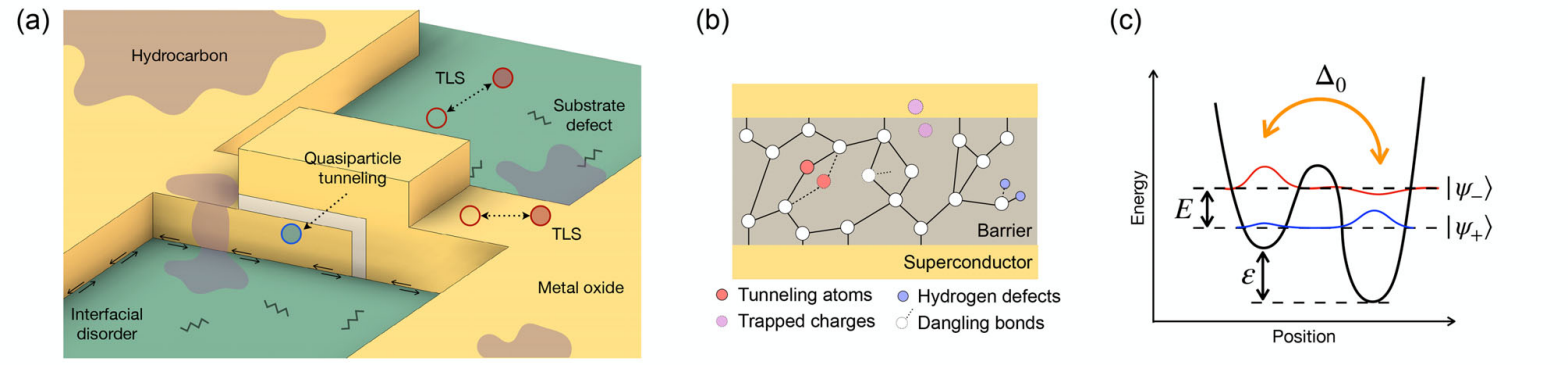
Energy loss sources around the junction and double‐well potential model of two‐level systems (TLS). (a) Schematic illustration of dominant loss mechanisms surrounding a transmon qubit. Material imperfections, such as hydrocarbon residues, interfacial disorder, amorphous metal–oxide layers, and substrate defects, host TLS that interact with the electric field within the qubits and give rise to dielectric loss. Additional losses arise from quasiparticle tunneling across the junction [128]. (b) Microscopic depiction of two‐level systems within the tunnel barriers between the superconducting electrodes [61, 129]. Fluctuations in their configurations induce qubit‐frequency noise, spectral diffusion, and relaxation hot spots. (c) Double‐well potential representation of a TLS in the standard tunneling model. An atom or atomic configuration occupies two nearly equivalent minima separated by a barrier, allowing quantum tunneling between localized states at low temperatures. This tunneling forms hybridized eigenstates $|\psi_{+}\rangle$ and $|\psi_{-}\rangle$ with a finite energy splitting determined by the asymmetry between the wells and the tunneling strength. Such TLS defects can resonantly interact with a qubit and contribute to dielectric loss [130].

**FIGURE 5:**
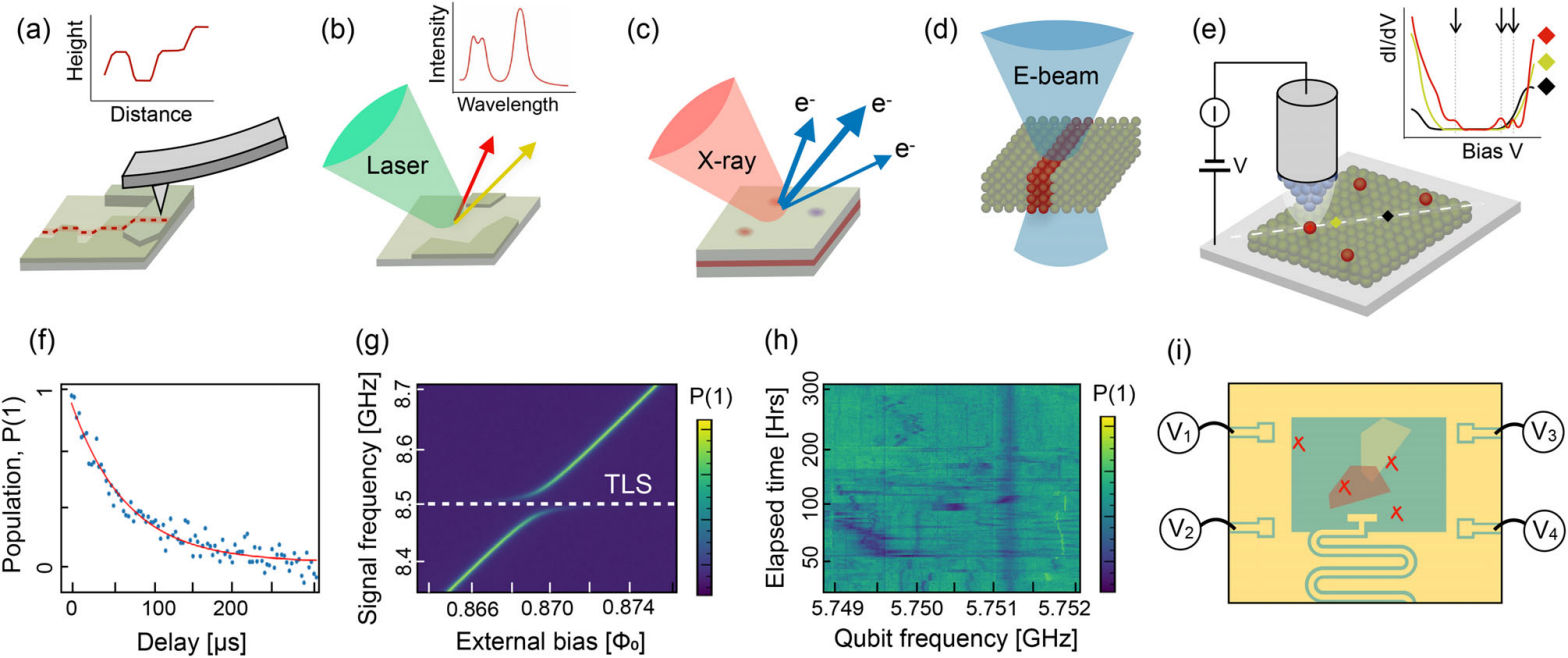
Material characterization for low‐loss Josephson junctions (JJs) and two‐level systems (TLS) evaluation of superconducting qubits. (a) Atomic force microscopy (AFM) for probing surface morphology, roughness, and thickness uniformity. (b) Raman and photoluminescence (PL) spectroscopies for probing layer number, strain, doping, phase purity, and transfer‐induced disorder. Red and yellow arrows represent scattered and emitted light, respectively. (c) X‐ray photoelectron spectroscopy (XPS) for analyzing elemental composition, oxidation states, and stoichiometry. (d) Scanning transmission electron microscopy (STEM) for probing crystallinity, atomic structure, and interfacial cleanliness. Electron energy‐loss spectroscopy (EELS) and energy‐dispersive spectroscopy (EDS) can be employed to map elemental distributions. (e) Scanning tunneling microscopy (STM) and scanning tunneling spectroscopy (STS) for atomic‐scale surface imaging and local density‐of‐states and defect characterization. (f) Qubit energy relaxation measurement (population P(1) versus delay) for extracting the energy relaxation time $T_{1}$ and assessing dielectric loss. (g) Qubit spectroscopy for revealing avoided level crossings arising from resonant coupling to a TLS. (h) Time‐dependent qubit spectroscopy for tracking TLS frequency fluctuations and spectral diffusion. (i) Device schematic illustrating electrical gate biasing and resonator‐based microwave characterization for identifying defect‐induced loss channels [159].

### 3.2 Energy Dissipation at the Junctions

#### 3.2.1 Two‐Level Systems in Tunnel Barriers

Solid‐state defects, commonly referred to as “two‐level systems” (or TLS), have garnered attention as a critical factor affecting the performance of superconducting qubits (Appendix D). Their low‐temperature behavior has long been a fascinating topic in condensed matter physics [113]. Although a universal consensus on their microscopic origins remains elusive, the characteristics observed in glassy materials are often attributed to the existence of two‐level systems (TLS), which are linked to the absence of long‐range atomic order in the amorphous state of these materials, as shown in Figure 4a,b.

Surprisingly, TLS display remarkable similarities across different chemical compositions and material arrangements [113, 131, 132]. These properties are generally explained by the standard tunneling model, described in Figure 4c [130]. In this model, the two energetically similar states of a TLS are separated by a finite potential barrier, forming a double‐well potential, whose Hamiltonian can be written as $H_{TLS}=\frac{1}{2}\varepsilon \sigma_{z}+\frac{1}{2}\Delta_{0}\sigma_{x}$, with an eigenstate splitting $E_{\mathrm{TLS}}=\sqrt{\varepsilon^{2}+\Delta_{0}^{2}}$. At sufficiently low temperatures, thermal activation between the two localized states is suppressed, and quantum tunneling hybridizes them into eigenstates split by $E_{\mathrm{TLS}}$. These defects can then exchange energy with electric fields, phonons, other TLS within the surrounding materials, or the host qubit, thereby contributing to dielectric loss, dephasing, and spectral diffusion [118, 133, 134, 135, 136].

TLS exhibit a distinct signature that provides valuable insights into their behavior [113]. For instance, their spectral signatures become thermally broadened or less distinct at elevated temperatures and energies [137] and are tunable with external factors like electric fields [61, 129, 138] and strain [128]. In a superconducting qubit's dressed frame, TLS can couple linearly or nonlinearly [139], offering a unique opportunity to probe their spectral distribution and distinguish TLS‐induced loss from other dissipation sources [108, 140]. These signatures enable quantitative studies of how TLS limit qubit coherence and provide experimental handles for identifying and mitigating defect‐induced loss.

TLS are pervasive in solid‐state devices, appearing in regions such as the bulk substrate, amorphous surfaces, interfaces, and within tunnel junction barriers [62, 111]. By transforming the transmon into a spectral TLS sensor [141], recent experiments have combined strain tuning and electric fields as control knobs to pinpoint the locations of TLS and their coupling strengths to the host qubit [59, 61, 142]. In addition, varying the spatial sensitivity of TLS in multimode planar resonators and transmons has enabled researchers to separate these components' contributions across the device [62, 143]. Measurements at different temperatures [137] and driven frames [139, 144] can also distinguish normal TLS from other effects, such as quasiparticle loss [140].

Research and development in JJ materials and superconducting qubit circuits have primarily focused on reducing TLS levels in amorphous bulk dielectric layers. For example, introducing Ta electrodes or annealed‐sapphire substrates has significantly mitigated bulk dielectric losses. Notable advances include identifying surface oxides that form in various superconducting materials and correlating these findings with different substrate purification processes [10, 62, 111, 137, 145, 146, 147, 148].

In addition to electric‐dipole coupling, some defects in bulk crystalline dielectrics may couple to qubits through strain‐ or piezoelectric‐mediated channels [149]. TLS can also form from adsorbates, leading to pure dephasing in flux‐tunable devices. Moreover, TLS at the junction interface are strongly coupled to the host qubit because the transmon geometry concentrates the electric field across the junction [62, 150, 151]. Therefore, fluctuating TLS within the tunneling barrier and surrounding dielectric can substantially vary qubit performance [62], contributing to device‐to‐device variability and temporal instability [3].

As several other decoherence channels have been progressively reduced in leading transmon platforms, TLS‐resilient JJ materials have become increasingly important as quantum processors scale up. Compared to amorphous materials, the dielectric loss (tanδ) of crystalline materials like sapphire [62, 149] has inspired efforts to develop crystalline tunnel barriers. One promising approach is to grow a thin epitaxial insulating layer, such as ${\mathrm{Al}}_{2}O_{3}$ [53, 152, 153, 154] or nitride‐based insulators [52, 54, 55, 155, 156, 157], between superconducting electrodes made from compatible materials. Preliminary TLS spectroscopy experiments have shown a reduced density of TLS in junctions [53, 158].

#### 3.2.2 Material‐Oriented Solutions and Their Characterization

Over the past decade, substantial effort has been devoted to identifying material platforms capable of surpassing the performance of aluminum‐based transmon qubits. A notable advance has been the widespread adoption of tantalum (Ta) shunt electrodes combined with Al‐based JJs, enabling energy relaxation times approaching $T_{1}∼1\;\mathrm{ms}$ [10, 146]. This improvement is largely attributed to Ta's chemically stable surface oxides, which exhibit significantly lower dielectric loss than those of aluminum or niobium [60].

While mitigating losses in the shunt electrodes has yielded impressive gains, attention has increasingly shifted to losses originating in the JJ itself, particularly in the amorphous ${\mathrm{AlO}}_{x}$ tunnel barrier. The strong electric‐field concentration within this amorphous layer enhances coupling between the transmon and parasitic TLS residing in ${\mathrm{AlO}}_{x}$. These strongly coupled TLS can stochastically drift into resonance with the qubit, producing sudden frequency shifts or energy relaxation “dropouts” that degrade device stability and necessitate frequent recalibration of quantum processors [3]. Consequently, further scaling of superconducting quantum processors will critically depend on strategies that reduce loss and variability associated with the junction itself.

One promising route to low‐loss JJs is using two‐dimensional (2D) van der Waals (vdW) materials [56, 66, 67, 68, 160]. 2D vdW materials embody a materials‐by‐design paradigm, enabling LEGO‐like modular assembly through the vertical stacking of atomically thin layers. In 2D vdW heterostructures, atomically thin crystals are held together by vdW forces rather than by covalent bonds. Compared with conventional amorphous‐barrier junctions, 2D vdW heterostructures may offer cleaner and more structurally well‐defined interfaces, potentially reducing some of the disordered motifs associated with TLS formation. Because the constituent crystals have atomically abrupt surfaces with few dangling bonds, they can, in principle, be assembled without the lattice‐mismatch constraints that often complicate conventional epitaxy [161]. A recent review further highlights the opportunities of 2D vdW JJs for crystalline tunnel barriers, symmetry engineering, moiré physics, and topological phenomena [56]. At the same time, transfer residues, trapped bubbles, edge disorder, and local thickness nonuniformity can still arise depending on fabrication techniques and thus remain important practical concerns.

Furthermore, researchers can systematically tune the Josephson energy by controlling both the tunneling‐barrier thickness and the junction area in 2D vdW heterostructures, providing a robust means to achieve target qubit frequencies. The diversity of available 2D vdW materials, combined with the freedom to engineer stacking sequences and twist angles, offers rich opportunities to implement JJs and qubits based on 2D vdW materials. Beyond low‐loss JJs, 2D vdW materials and their heterostructures offer several compelling advantages for superconducting qubits.

To translate the putative material advantages into genuinely low‐loss JJs, the interfaces must be verified to be clean, abrupt, and structurally uniform across multiple length scales. Figure 5a–e summarizes several characterization techniques for evaluating materials for low‐loss JJs, which can be applied to various materials, including 2D vdW materials and their heterostructures [159, 162]. Optical microscopy provides a rapid first screen for cracks, trapped bubbles, transfer residues, and macroscopic nonuniformity, whereas atomic force microscopy (AFM) quantifies thickness, roughness, step morphology, and nanoscale bubbles that often signal interfacial contamination. For 2D vdW superconductors and insulators, Raman and photoluminescence (PL) spectroscopy can further provide non‐destructive information on layer number, strain, doping, phase purity, and transfer‐induced disorder. X‐ray photoelectron spectroscopy (XPS) and, where necessary, ultraviolet photoelectron spectroscopy or secondary‐ion mass spectrometry are particularly valuable for identifying oxidation states, stoichiometry, adsorbates, and interdiffusion at the JJ interfaces. At higher spatial resolution, cross‐sectional scanning transmission electron microscopy (STEM) combined with electron energy‐loss spectroscopy (EELS) or energy‐dispersive X‐ray spectroscopy (EDS) directly reveals barrier‐thickness uniformity, lattice order, edge damage, interfacial abruptness, and any residual amorphous layers, while scanning tunneling microscopy/spectroscopy (STM/STS) can resolve atomic‐scale defects and the local density of states that may seed subgap states or TLS. In this sense, these tools are not merely descriptive material probes; rather, they test the central premise of low‐loss JJs, namely that the relevant tunneling interfaces can be engineered to avoid the structurally and chemically disordered motifs that dominate dielectric loss in conventional amorphous‐barrier junctions.

Once materials are incorporated into JJs or superconducting qubits, additional characterization tools can directly evaluate device performance, as shown in Figure 5f–i. At the JJ level, low‐temperature transport measurements, such as I–V characteristics for critical current, normal‐state resistance, subgap resistance, excess current, switching‐current statistics, and magnetic‐field interference patterns, provide an important bridge between ex situ materials analysis and circuit performance by testing the uniformity of tunneling and screening for pinholes, edge‐dominated transport, or parasitic dissipative channels. At the qubit level, coherence measurements become the most relevant figure of merit since qubit coherence represents a comprehensive measure of the lifetime of stored quantum information. Although the energy relaxation time $T_{1}$ is most commonly reported, the phase coherence time $T_{2}$ is equally important because it reflects sensitivity to fluctuating charges, critical‐current noise, flux noise, and broader electromagnetic instability in the junction interface and its environment. At the same time, a single coherence time at a single operating frequency should not be overinterpreted, since TLS can be spectrally distributed or localized, and temporally intermittent or fluctuating, their influence can easily be underestimated or overinterpreted within a limited measurement window. A more practical approach is therefore to map coherence over a wide frequency range, either by explicit flux or gate tuning or by exploiting AC Stark shifts in fixed‐frequency qubits [61, 138, 141]. When this frequency‐resolved characterization is extended over time, it becomes TLS spectroscopy [57, 141, 163], enabling extraction of TLS spectral density, coupling strength, and switching dynamics, and converting qubit coherence from a simple benchmark into a quantitative diagnostic of material quality [164].

Beyond a single JJ, TLS‐sensitive qubit architectures further amplify microscopic information about loss. Qubits with multiple JJs increase the effective number of participating defects and can therefore enhance sensitivity to weak, distributed dissipation channels. Likewise, fluxonium and related circuits [11, 115, 165, 166, 167, 168, 169], with their intrinsically broad transition spectra and strong anharmonicity, provide a particularly powerful platform for probing defect dynamics over a wide energy range. For qubits based on low‐loss JJs, the key performance target is therefore not simply a high average $T_{1}$, but a pronounced suppression of the spectral density of strongly coupled TLS, together with reduced temporal drift, fewer relaxation hot spots, and smaller device‐to‐device variability. Demonstrating such suppression would provide direct evidence that the material and interface engineering strategy has addressed the dominant microscopic loss channels, thereby establishing these junction platforms as credible building blocks for scalable, low‐loss superconducting quantum processors.

#### 3.2.3 Quasiparticle Tunneling

The elementary excitations of a superconductor, known as *quasiparticles*, arise from breaking Cooper pairs in the superconducting condensate [106]. Such excitations can be generated by impacts from high‐energy particles, which deposit energy exceeding the superconducting gap [170]. If the superconducting‐gap asymmetry across the two electrodes is smaller than the qubit transition energy, a nonequilibrium quasiparticle can absorb energy from the qubit while tunneling across the JJ interface, thereby inducing qubit relaxation [63]. For superconducting quantum processors, impacts from high‐energy particles [171] or infrared photons [107] generate sudden spikes in quasiparticle density that propagate across the processor [172], leading to catastrophic correlated errors that are challenging to account for using existing quantum error correction codes [63, 170].

Recent studies highlight that gap engineering mitigates this process by creating a sufficiently large gap mismatch across the junction, making quasiparticle tunneling, accompanied by qubit‐energy absorption, energetically unfavorable [63, 64, 65, 69]. This technique increases the disparity in superconducting gaps across the JJ interface, keeping the qubit transition frequency below the gap difference across the junction and suppressing resonant energy exchange between a quasiparticle and the qubit. In thin superconducting films, where the film thickness approaches the penetration depth, the superconducting gap becomes highly sensitive to thickness variations [65]. By fabricating JJs with electrodes of varying thickness, one can optimize the gap difference across the junction and protect against quasiparticle tunneling.

Experimental results show significant improvements in qubit coherence under high‐energy impact conditions when qubits are designed with large gap differences between junction electrodes [63]. This approach paved the way for more effective error reduction in surface‐code implementations [91]. Future efforts to develop novel JJ materials would benefit from adapting gap engineering to minimize quasiparticle losses and their detrimental effects.

### 3.3 In Situ Tunability

Tuning the spectrum of superconducting qubits is crucial for flexibility and control, helping alleviate spectral crowding, mitigate frequency collisions with TLS, and enable the engineering of entangling interactions between multiple qubits. Tunability in qubits is typically implemented by threading magnetic flux through flux‐sensitive qubits, such as split‐junction transmons [117], flux qubits [173] (Figure 6a), or fluxoniums [165]. However, driving multiple flux‐tunable qubits often leads to structural complexity and conflicting requirements. For instance, processor components can form large superconducting loops through the ground plane, thereby increasing sensitivity to flux noise and introducing parasitic coupling and crosstalk. The current required for flux control imposes a significant thermal load on a cryostat, potentially impeding scalability. Furthermore, stray currents from the ground plane are sensitive to wire bonds or air bridges, potentially leading to inaccurate flux biasing or uncontrolled parameter offsets. Therefore, various qubit tuning strategies must be explored to improve the performance and scalability of superconducting quantum processors.

**FIGURE 6:**
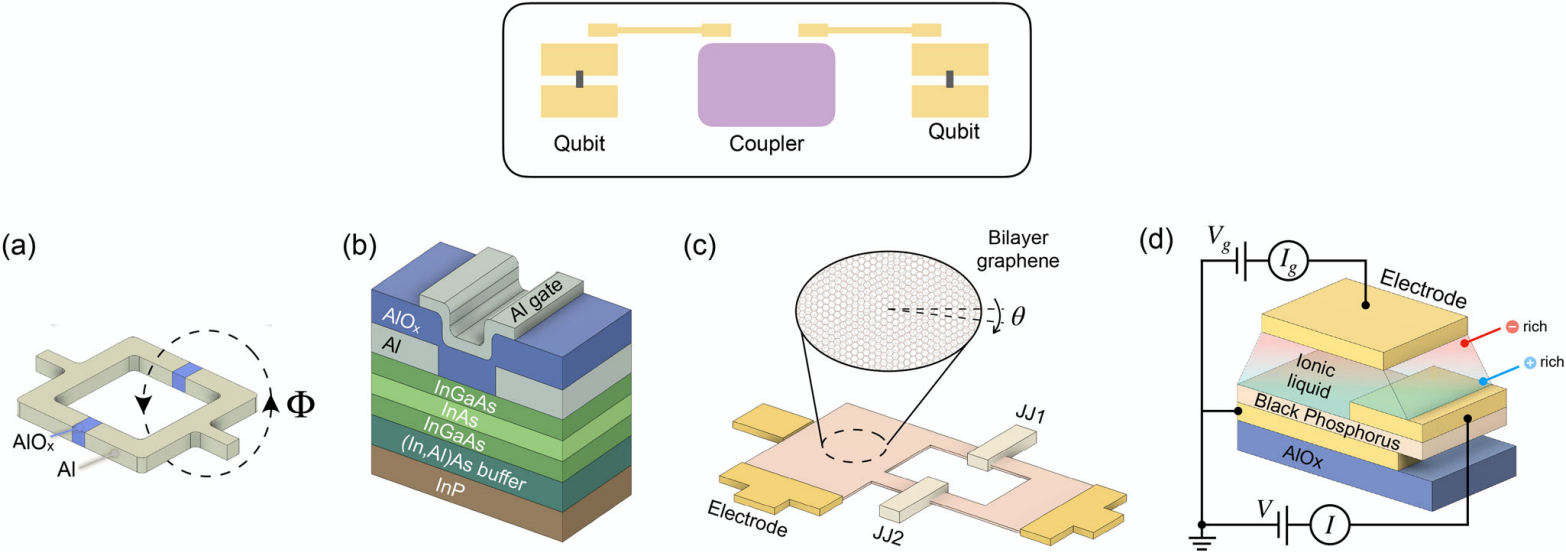
In situ‐tunable Josephson junction (JJ) platforms for superconducting qubits. (a) Al/${\mathrm{AlO}}_{x}$/Al superconducting quantum interference device (SQUID) in which magnetic flux Φ tunes the effective Josephson energy. Beyond flux qubits [173], flux‐controlled junctions are commonly used in tunable couplers to achieve switchable interactions between superconducting qubits. (b) Epitaxial Al/two‐dimensional electron gas (2DEG) planar JJ formed on a semiconductor heterostructure, enabling gate control of the critical current through a highly transparent superconductor (SC)/semiconductor (SM) interface, making it attractive for scalable gatemon qubits [16, 72, 73] and voltage‐tunable couplers [12, 188, 189, 190, 191]. (c) Monolithic SQUID in magic‐angle twisted bilayer graphene with two independently gated weak links, JJ1 and JJ2. Local electrostatic control tunes the critical current in each arm, enabling asymmetric or symmetric SQUID operation and phase‐sensitive measurements of the twisted‐graphene weak links [75]. (d) Ionic‐liquid‐gated vertical Nb/black‐phosphorus/Nb Josephson field‐effect transistor (FET). Electrostatic gating modulates the critical current and junction resistance in an atomic‐scale vertical channel, consistent with strongly inhomogeneous, edge‐ or corner‐dominated supercurrent transport in the black‐phosphorus barrier [77].

An alternative to flux‐tuning is voltage‐tuning. In superconducting qubits, the presence of a superconducting island introduces a charge degree of freedom, which allows for voltage control. In practice, this sensitivity is strongly suppressed in the transmon regime. The transmon, for instance, can be made charge‐sensitive by reducing the $E_{J}/E_{C}$ ratio, gradually mapping to the charge qubit. At the same time, charge‐sensitive qubits are highly susceptible to the ubiquitous environmental charge noise, which severely degrades coherence times. In contrast, charge‐sensitive superconducting elements are well‐suited as couplers, as they mediate interactions between qubits rather than storing quantum information themselves. As a result, their increased sensitivity to charge noise is less detrimental. Moreover, voltage‐tunable couplers can avoid the aforementioned issues that arise for flux‐tunable circuits.

Couplers require strong frequency tuning to implement gates while also turning off qubit interactions. More fundamentally, the achievable tuning range in charge‐sensitive superconducting elements is ultimately limited by the charging‐energy scale $E_{C}$, which cannot easily exceed several gigahertz due to stray/coupling capacitances. An alternative to conventional Al/${\mathrm{AlO}}_{x}$/Al JJ‐based transmon qubits is the gatemon qubit, in which the Josephson energy is controlled electrostatically through a semiconductor weak link. This enables qubit frequency tuning via gate voltage rather than magnetic flux. Gatemon qubits have been realized using semiconducting nanowire‐based JJs [70, 174, 175], two‐dimensional electron gas (2DEG) systems [16, 176, 177, 178, 179], and 2D vdW JJs [74, 75, 76, 180, 181, 182, 183, 184, 185, 186, 187]. Semiconductor‐based gatemon platforms, particularly nanowire‐based JJs and 2DEG systems, have demonstrated multi‐gigahertz frequency tuning, with coherence times reaching the 20 µs range, whereas implementations of 2D vdW JJs are at an earlier stage. While voltage tunability can, in principle, be used for qubits, its most compelling near‐term role is in tunable couplers, where charge‐noise sensitivity is less detrimental. For coupler applications, 2DEG systems and 2D vdW JJs are particularly promising, as their planar geometries can relax some of the dimensional constraints that limit the achievable $E_{J}$ in semiconducting nanowire‐based JJs. In the following, we outline a platform for fundamental quantum device investigations that offers viable routes toward voltage‐tunable superconducting circuits.

#### 3.3.1 Gate‐Tunable 2DEG Systems

When a semiconductor is coupled to superconducting leads, superconducting correlations are induced into the semiconductor via the proximity effect, enabling dissipationless transport through an otherwise nonsuperconducting material [192, 193]. Microscopically, the proximity effect arises from Andreev reflection at the SC/SM interface, where an electron incident from the semiconductor is retroreflected as a hole while a Cooper pair is transferred into the superconductor. As discussed in Section 2, coherent Andreev processes across the junction give rise to discrete ABS within the superconducting gap, which carry the Josephson supercurrent (Equation 5). The ABS energies depend on the superconducting phase difference across the junction and on the number and transparency of the conduction channels. In SC/SM JJs, electrostatic gating directly controls channel transparency by changing the semiconductor carrier density and chemical potential, enabling in situ tuning of the Josephson current and the effective Josephson energy $E_{J}$.

2DEG systems are widely used for implementing SC/SM JJs and offer a potentially scalable platform. A representative example is the epitaxial growth of an Al/InGaAs/InAs/InGaAs heterostructure on an InP substrate, as shown in Figure 6b. In 2DEG systems, band engineering positions the Fermi level within the conduction band of a few‐nanometer‐thin InAs quantum well, confining carriers to a 2DEG. This is particularly well‐suited for gate‐defined JJs due to its high carrier mobility, which enables ballistic transport and channel transparency. Moreover, epitaxial Al/InAs junctions form clean interfaces and highly transparent contacts, a prerequisite for the strong superconducting proximity effect [71, 194]. The quality of SC/SM interfaces in 2DEG systems has been experimentally verified with multiple Andreev reflections [73].

Leveraging these advances, InAs‐based gatemon qubits have been realized, exhibiting energy relaxation times of $T_{1}∼1\;\mu s$ and coherence times of $T_{2}∼2\;\mu s$ [16]. More recently, alternative material platforms have also been explored, including planar Ge‐based gatemon qubits, which are still at an early stage and currently exhibit $T_{1}∼100\;\mathrm{ns}$ [177, 178]. Importantly, multiple studies indicate that the semiconductor weak link itself is not the dominant source of decoherence in gatemon devices. Instead, loss mechanisms are primarily attributed to the limited quality factor of the Al film, dielectric loss from thick ${\mathrm{AlO}}_{x}$ layers used for top electrostatic gates, and bulk substrate loss originating from the InP substrate. These limitations can be mitigated through materials and architectural optimization, such as increasing the Al thickness to reduce inductive loss, employing low‐loss gate dielectrics (e.g., h‐BN), or wafer‐bonding the heterostructure to a low‐loss substrate [71].

Continued improvements along these directions may enable semiconductor‐based gatemon coherence times to exceed 10μs. While this remains below the state‐of‐the‐art coherence achieved in transmon qubits, such performance is sufficient to enable gatemon‐based tunable couplers [12, 188, 189, 190, 191]. In this architecture, two transmon qubits are coupled via an SC/SM JJ whose transparency is dynamically controlled using voltage pulses, effectively switching the interaction on at finite transparency and off at near‐zero transparency. Theoretical studies have suggested that, under favorable assumptions, two‐qubit gates may be implemented on ∼50ns timescales with coherent error rates below 0.01% [12].

Further performance gains may be achieved by integrating gatemon‐based couplers into a flip‐chip architecture [177, 188]. In such designs, through‐silicon vias (TSVs) can be used to redirect the electromagnetic mode of the gatemon away from the lossy InP substrate and into a low‐loss Si interposer, thereby suppressing substrate‐induced loss. TSVs additionally provide electromagnetic shielding, reducing crosstalk between circuit elements. Notably, although transmon qubits inherit some decoherence from the coupler, it has been shown that a coupler with $T_{2}∼1\;\mu s$ can still support transmon coherence times exceeding 100μs while enabling fast, high‐fidelity gates [188].

Beyond tunable couplers, a variety of alternative coupling schemes based on gatemons have been proposed. For example, an SC/SM junction can be embedded in a coplanar waveguide to realize a voltage‐controlled superconducting quantum bus [72, 189]. Alternatively, the junction may function as a gate‐tunable capacitor, enabling coupling between qubits across the transition from a fully transmitting to a fully insulating regime [191]. Finally, hybrid architectures combining gatemon qubits with transmons enable parametric entangling gates driven by voltage modulation, offering additional flexibility for scalable superconducting quantum processors [190].

#### 3.3.2 Gate‐Tunable 2D vdW JJs

Gate‐tunable 2D vdW JJs offer a promising route to electrically reconfigurable superconducting circuits while retaining their material advantages, as described in Section 3.2.2. Such superconducting qubits may benefit from potentially low‐loss, atomically clean interfaces, as well as the large electrostatic tunability of 2D vdW materials and their heterostructures.

Graphene was among the first 2D materials used as a weak link in 2D vdW JJs [74, 180, 181, 183, 195, 196]. Similar to SC/SM JJs in 2DEG systems, the supercurrent in graphene‐based JJs is driven by ABS. Graphene provides high‐transparency conduction channels due to its high mobility and ballistic transport, while its low density of states may reduce the sensitivity of junction properties to gate‐voltage fluctuations. Electrostatic gating further shifts the Fermi energy, altering the carrier density and providing direct control over the junction critical current and the Josephson energy $E_{J}$. Gate‐dependent microwave spectroscopy has provided clear evidence of ballistic transport and high‐quality interfaces between graphene and superconducting contacts [181]. Several groups have demonstrated voltage‐tunable graphene‐based qubits with frequency shifts spanning multiple gigahertz, with the tunability determined by the gate geometry and dielectric thickness [74, 180, 196]. Notably, the qubit transition frequency often shows a non‐monotonic, oscillatory dependence on gate voltage, creating multiple operational sweet spots. These sweet spots may reduce sensitivity to gate‐induced charge noise, thereby improving coherence.

Notably, coherent control of graphene‐based gatemon qubits has been demonstrated, with reported coherence times on the order of 50 ns [74]. These relatively short coherence times have been attributed, at least in part, to radiative losses through the gate electrode and fabrication‐related imperfections in 2D vdW JJ‐based qubits. Radiative losses can be reduced through improved circuit design, for example, by further decoupling the gate electrode from the qubit mode and exploiting symmetry to suppress coupling to the transmon degree of freedom. In addition, integrating low‐pass filters on the gate line can attenuate noise at the qubit frequency. It is worth noting that available evidence suggests that the presently observed coherence times are likely substantially influenced by circuit‐level loss channels beyond the graphene weak link itself. Future experiments incorporating conventional tunnel‐junction transmons or coplanar waveguide resonators on the same chip as graphene‐based qubits could help disentangle junction‐associated losses from those arising in shunt capacitors, substrates, or control circuitry. The microwave characterization techniques discussed in Section 3.2.2 may also provide insight into TLS densities associated with graphene junctions.

Various 2D vdW materials offer complementary approaches for realizing gate‐tunable JJs and have demonstrated compatibility with elements required for superconducting circuit integration, including magic‐angle twisted bilayer graphene [75, 182] (Figure 6c), ${\mathrm{MoS}}_{2}$ [197], ${\mathrm{MoTe}}_{2}$ [198], ${\mathrm{Bi}}_{2}O_{2}\mathrm{Se}$ [199], and black phosphorus [77] (Figure 6d). For example, electrostatic gates can define superconducting leads and non‐superconducting junction regions within magic‐angle twisted bilayer graphene, enabling gate‐defined JJs that exhibit both DC and AC Josephson effects while largely avoiding external interfaces caused by multi‐material weak links [182]. Building on this concept, a monolithic SQUID based on magic‐angle twisted bilayer graphene with two locally tunable weak links has demonstrated phase‐coherent interference, symmetric and asymmetric SQUID operation, direct access to the current‐phase relation, and large gate‐tunable kinetic inductances reaching the μH scale [75]. More broadly, twisted graphene superconductors can also serve as circuit elements with unusually large, electrostatically tunable kinetic inductance. For example, twisted tri‐layer graphene weak links in a SQUID geometry exhibit kinetic inductances up to ∼150nH/□ and coherence lengths approaching ∼200nm [187]. Few‐layer black phosphorus has been used as the vertical weak link in an ionic‐liquid‐gated Nb/black‐phosphorus/Nb Josephson field‐effect transistor (FET), in which the critical current and junction resistance are strongly gate tunable despite the ultrashort transport length, consistent with a strongly inhomogeneous supercurrent flow, possibly dominated by edge or corner conduction paths in the BP barrier [77]. These results show that 2D vdW materials and their heterostructures are not only compatible with superconducting weak‐link architectures, but can also support monolithic, phase‐coherent, and vertical Josephson devices with strong in situ electrical control.

In the context of tunable couplers, 2D vdW JJs can be integrated using strategies similar to those developed for flux‐tunable couplers and SC/SM junctions. Because both platforms support gate‐tunable transparency and ABS‐mediated supercurrents, they can support analogous coupler concepts. However, 2D vdW materials offer gate‐tunable superconductivity within a single material, unlike conventional SC/SM JJ‐based 2DEG systems. A particularly compelling example is monolayer ${\mathrm{WTe}}_{2}$, a 2D vdW topological insulator that becomes superconducting upon gating [200, 201]. In its pristine form, ${\mathrm{WTe}}_{2}$ is a bulk insulator with conducting edge modes characteristic of quantum spin Hall insulators [202]. Upon gating, ${\mathrm{WTe}}_{2}$ undergoes a transition to a superconducting phase at remarkably low carrier densities, the mechanism for which remains an open question. By patterning multiple gate electrodes across a single ${\mathrm{WTe}}_{2}$ monolayer, JJs can be defined in situ, enabling continuous tuning between fully insulating and fully superconducting regimes. Experimental observations of both superconductivity and the Josephson effect in gated ${\mathrm{WTe}}_{2}$ have already been reported [76]. Integrating this material platform into superconducting quantum processors could open intriguing opportunities for realizing tunable couplers with exceptionally large on/off ratios, enabled by the ability to electrostatically switch superconductivity itself rather than merely modulating junction transparency.

### 3.4 Device Footprint

Utility‐scale, fault‐tolerant quantum computers will likely require millions of physical qubits. While the transmon has been foundational for superconducting quantum processors [90, 91, 117, 124], its large footprint due to the shunt capacitor is a bottleneck to scaling. Typical planar transmons often occupy footprints exceeding $0.1\;{\mathrm{mm}}^{2}$, which becomes increasingly problematic as chip area, interconnect density, packaging complexity, and cryogenic overhead all tighten with scale [146, 206]. The central challenge is that reducing the qubit footprint generally increases the electric‐field participation of the junction barrier and nearby dielectrics. Compact qubit architectures are therefore attractive only insofar as these materials remain sufficiently low‐loss and uniform. Recent efforts therefore aim to miniaturize superconducting qubits while preserving their intrinsic resilience to charge noise, for example, through 2D vdW shunt capacitors or advanced fabrication strategies [14, 78, 79, 80, 124, 207].

2D vdW materials naturally enable compact capacitive elements through parallel‐plate geometries and, more broadly, a range of compact JJ and qubit platforms [208]. Representative examples include ${\mathrm{NbSe}}_{2}$/${\mathrm{NbSe}}_{2}$ [203] (Figure 7a), ${\mathrm{NbSe}}_{2}$/h‐BN/${\mathrm{NbSe}}_{2}$ [14, 78] (Figure 7b), ${\mathrm{NbSe}}_{2}$/${\mathrm{WSe}}_{2}$/${\mathrm{NbSe}}_{2}$ [15, 208], and h‐BN‐encapsulated graphene JJs [74]. As shunt dielectrics, 2D vdW insulators such as h‐BN can enable compact parallel‐plate capacitors with low loss. For example, a monolayer of h‐BN is only ∼0.3 nm thick, much thinner than conventional amorphous dielectric layers used in superconducting circuits, enabling much higher capacitance per unit area, provided leakage, dielectric strength, and fabrication uniformity can be adequately controlled. Beyond h‐BN, high‐κ 2D vdW dielectrics with ultralow equivalent oxide thickness may offer an additional route to increasing capacitance density in compact junction architectures [81]. If such barriers can be integrated while maintaining sufficiently low dielectric loss, they could be attractive for merged‐element transmon (*mergemon*) designs [14, 78, 204].

**FIGURE 7:**
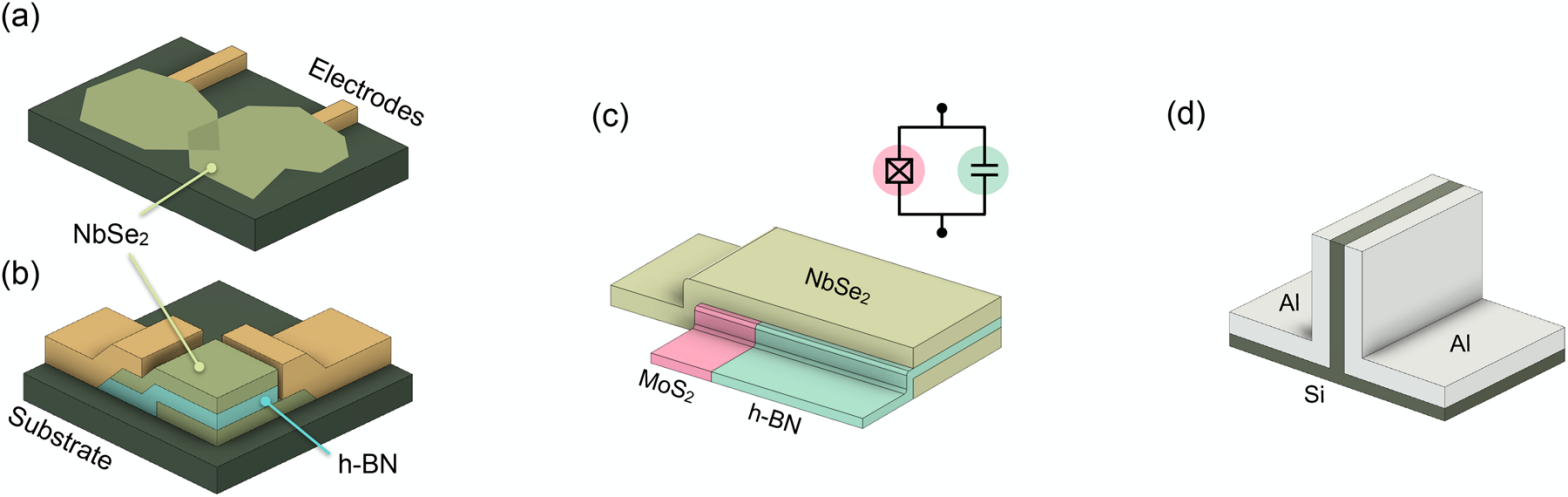
Compact qubit designs. (a) ${\mathrm{NbSe}}_{2}$/${\mathrm{NbSe}}_{2}$ two‐dimensional van der Waals (2D vdW) Josephson junction (JJ) with a vacuum gap, providing an oxide‐free weak link that avoids an amorphous interfacial barrier [203, 204, 205]. (b) ${\mathrm{NbSe}}_{2}$/h‐BN/${\mathrm{NbSe}}_{2}$ 2D vdW shunt capacitor with a low‐loss h‐BN dielectric interlayer, offering a promising approach to realize high‐coherence superconducting qubits with small layout geometries [14, 78]. (c) A promising conceptual architecture for a highly coherent yet compact 2D vdW transmon qubit, consisting of ${\mathrm{NbSe}}_{2}$ electrodes, a small‐area ${\mathrm{MoS}}_{2}$ (or ${\mathrm{WSe}}_{2}$) weak link, and an adjacent h‐BN dielectric. (d) A silicon‐based, fin‐shaped merged‐element transmon (FinMET) with high fabrication precision, compactness, and CMOS compatibility [79].

Furthermore, as tunnel barriers or weak‐link materials, 2D vdW semiconductors such as ${\mathrm{MoS}}_{2}$ and ${\mathrm{WSe}}_{2}$ have been reported to exhibit lower device‐to‐device variation in the resistance‐area (RA) product than conventional amorphous oxides, consistent with improved barrier uniformity and potentially cleaner interfaces [15, 197]. 2D vdW JJs with semiconducting tunnel barriers, for example, a ${\mathrm{NbSe}}_{2}$/${\mathrm{WSe}}_{2}$/${\mathrm{NbSe}}_{2}$ heterostructure [15, 208], could provide a route toward more compact JJs and, if low‐loss interfaces are maintained, improved qubit coherence. Recent ${\mathrm{MoS}}_{2}$‐based implementations have primarily focused on flux‐tunable SQUID‐type qubits, where coherence may also be influenced by vortex trapping in the junction and readout circuitry [15, 197]. In this context, as shown in Figure 7c, a 2D vdW JJ consisting of ${\mathrm{NbSe}}_{2}$ electrodes, a small‐area ${\mathrm{MoS}}_{2}$ (or ${\mathrm{WSe}}_{2}$) weak link, and an adjacent h‐BN dielectric may be a promising architecture for a highly coherent yet compact 2D vdW transmon qubit. In this conceptual architecture, the h‐BN layer serves as a JJ‐embedded shunt dielectric, while the ${\mathrm{MoS}}_{2}$ (or ${\mathrm{WSe}}_{2}$) layer serves as the weak‐link tunnel barrier, thereby enabling supercurrent tuning.

Another straightforward path to minimizing device footprint is to leverage the intrinsic capacitance of the JJ to eliminate or reduce the need for separate shunt capacitors in quantum circuits. Conventional mergemon qubits use a thicker oxide grown at higher oxidation pressures and a larger junction area [207]. This approach significantly improves scalability by reducing the footprint by roughly two orders of magnitude while maintaining competitive coherence times of $T_{1}∼100\;\mu s$ and $T_{2}∼50\;\mu s$ through optimized thermal annealing processes [207], which can mitigate the associated junction loss. Other implementations, such as the silicon‐based fin‐shaped merged‐element transmons (FinMETs) shown in Figure 7d, further illustrate this approach through anisotropic etching and float‐zone silicon fabrication, which enable high precision and CMOS compatibility [79]. This compactness, however, increases electric‐field participation in the junction dielectric, so the success of merged‐element architectures depends critically on barrier quality, defect density, and processing‐induced disorder.

### 3.5 Noise‐Protected Qubits

The advancement of superconducting quantum processors over the past two decades has been driven primarily by the transmon qubit [90, 91, 117]. The simple structure of the transmon, involving only a JJ and a large shunting capacitor, eases fabrication, control, and scalability. However, the transmon's weak nonlinearity, due to its simple structure, limits its anharmonicity and does not protect against decoherence. These issues have become more apparent as the margin for error in high‐fidelity quantum gates has become tighter [209]. As a result, other superconducting circuits have been explored for enhanced anharmonicity and/or protection, often with increased circuit complexity [11, 82, 114, 210].

One promising approach to protecting quantum information is to leverage Cooper‐pair parity [82, 83]. In superconducting circuits with an exact or approximate Cooper‐pair‐parity symmetry, the Hilbert space separates into even‐ and odd‐charge sectors. When the dominant noise operators preserve this parity, matrix elements between the logical states can be strongly suppressed, reducing sensitivity to charge‐like decoherence channels. If the junction Hamiltonian is dominated by quartet tunneling rather than single‐Cooper‐pair tunneling, the two parity sectors can remain nearly decoupled, enabling a protected qubit manifold with enhanced anharmonicity and reduced sensitivity to charge‐like decoherence channels. Another envisioned route to hardware‐efficient fault tolerance leverages superconducting circuits with dual nonlinearities to host grid states that are intrinsically decoupled from dominant decoherence channels [211, 212, 213]. In these devices, parity and redundancy are implemented at the hardware level, providing built‐in error correction.

A Cooper‐quartet tunneling (CQT) JJ is required to realize a Cooper‐pair parity‐protected qubit. A CQT junction is effectively described by a dominant second‐harmonic Josephson term, corresponding to the coherent transfer of two Cooper pairs. As a result, states of even and odd Cooper‐pair numbers cannot be coupled by the tunneling of quartets and are disjoint. In addition, the CQT junction constitutes a key building block of the grid‐state qubit and underpins its intrinsic quantum error protection [214]. The effective CQT junctions have been demonstrated with circuits constructed with SC/SM junctions [84] and with Al/$\mathrm{Al}O_{x}$/Al junctions and superinductors [83, 215]. However, such schemes require external voltage or magnetic flux control, as well as stringent symmetry between the constituent elements. Alternatively, several materials that intrinsically favor CQT offer a complementary route in which the desired nonlinearity emerges directly from the junction's physics, without the need for external control parameters. Here, we explore how d‐wave SCs and π‐Josephson junctions can be integrated into superconducting processors to realize robust, noise‐protected qubits.

In this Subsection 3.5, we focus specifically on parity‐protected superconducting qubits, where protection arises from the engineered suppression of single‐Cooper‐pair tunneling and the dominance of higher‐order Josephson processes.

#### 3.5.1 d‐Wave Superconductors

d‐wave SC platforms can intrinsically suppress first‐order Josephson coupling and thereby favor CQT, depending on material symmetry and twist angle [17, 85, 86]. CQT in d‐wave platforms arises from the symmetry and sign structure of the d‐wave order parameter $\psi_{d}$, which undergoes a sign inversion $\psi_{d}\to -\psi_{d}$ under a $90^{\circ }$ rotation, analogous to the atomic d‐orbitals. This symmetry property can be leveraged in two ways. The first is to stack a d‐wave SC with an s‐wave SC. The Ginzburg–Landau free energy density for this configuration is given by

(9) $$\begin{array}{cc} F[\psi_{d},\psi_{s}] & =F_{d}\left[\psi_{d}\right]+F_{s}\left[\psi_{s}\right]+A|\psi_{d}{|}^{2}{\left|\psi_{s}\right|}^{2}\\ & \;+B\left(\psi_{d}\psi_{s}^{*}+c.c.\right)+C\left(\psi_{d}^{2}\psi_{s}^{*2}+c.c.\right), \end{array}$$

where $\psi_{d}\left(s\right)$ is the order parameter of the d(s)‐wave SCs and $F_{d}\left(s\right)$ is the free energy of each SC. A, B, and C represent couplings; in particular, B is the first‐order Josephson coupling for Cooper‐pair tunneling, and C is the second‐order Josephson coupling for CQT. If both SCs obey tetragonal symmetry, the free energy density is conserved under a $90^{\circ }$ rotation. However, this rotation leads to $\psi_{d}\to -\psi_{d}$, so the only way for $F[\psi_{d},\psi_{s}]$ to be invariant is for B=0. On the other hand, the CQT term C is unaffected. Therefore, the first‐order Josephson coupling is symmetry‐forbidden in the ideal limit, allowing the second‐order Josephson term to dominate in a d/s bilayer stack. Although early‐stage experiments on d/s bilayer stacks have been reported [216, 217, 218], evidence for CQT in such structures remains elusive. Advances in heterostructure fabrication and interface control can enable experimental demonstrations of this platform.

Another approach is to stack two d‐wave SCs with a relative twist angle in between. The free‐energy density is given by

(10) $$\begin{array}{cc} F[\psi_{1},\psi_{2}] & =F_{1}\left[\psi_{1}\right]+F_{2}\left[\psi_{2}\right]+A|\psi_{1}{|}^{2}{\left|\psi_{2}\right|}^{2}\\ & \;+B\left(\psi_{1}\psi_{2}^{*}+c.c.\right)+C\left(\psi_{1}^{2}\psi_{2}^{*2}+c.c.\right), \end{array}$$

where the subscripts denote the top and bottom d‐wave SCs. Twisting the second superconducting electrode from $0^{\circ }$ to $90^{\circ }$ results in $\psi_{2}\to -\psi_{2}$. To conserve the free energy density, the coefficient B must also change sign as the twist angle increases and crosses a node (B=0) at $45^{\circ }$. Therefore, twisted bilayers of d‐wave superconductors provide a natural route toward CQT‐dominated Josephson coupling. Experimental signatures consistent with dominant CQT or second‐harmonic Josephson coupling have been reported in 2D vdW twisted bilayer BSCCO, a high‐temperature d‐wave SC [219].

The next milestone is to shunt a d/s or d/d junction with a large capacitor, as shown in Figure 8a, to realize a protected superconducting qubit [17, 85]. Like the transmon, the large capacitive shunt suppresses charge noise and enhances phase coherence. Three criteria must be met for a successful implementation. First, a pristine, high‐transparency interface is required to maximize the desired quartet‐tunneling amplitude and minimize uncontrolled single‐pair processes. Independently, a sufficiently large shunt capacitance is needed to suppress charge dispersion and stabilize the protected qubit manifold.

**FIGURE 8:**
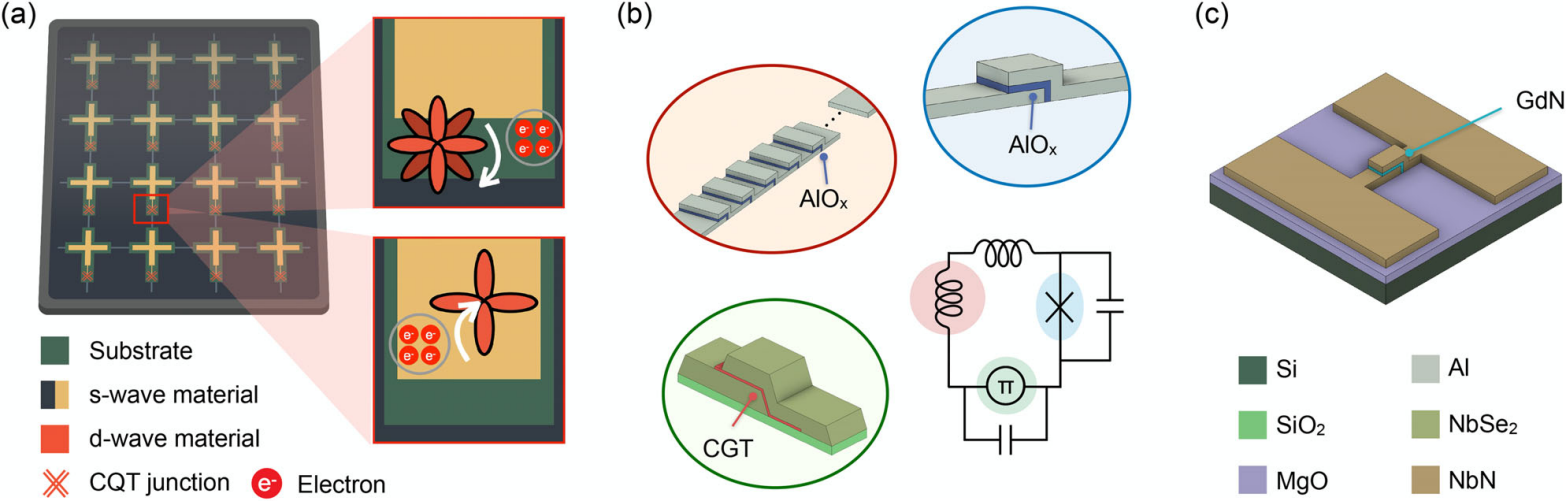
Parity protection with Cooper‐quartet tunneling (CQT) junctions. (a) Array of d‐wave Josephson junctions (JJs) with capacitive shunt for charge noise resilience. Two types of CQT junctions are shown, leveraging either stacking an s‐wave superconductor (SC) with a d‐wave SC [17] (lower) or a relative $45^{\circ }$ rotation between two d‐wave SCs [85, 86] (upper). (b) Noise‐resilient qubit realized in a rhombus circuit geometry. A ferromagnetic insulating ${\mathrm{Cr}}_{2}{\mathrm{Ge}}_{2}{\mathrm{Te}}_{6}$ (CGT) interlayer realizes a π‐JJ, eliminating the need for external flux biasing. Effective CQT emerges from the interplay between the single junctions and the surrounding Josephson‐junction arrays. (c) Intrinsic CQT in a GdN‐based π‐JJ. CQT can dominate over single‐Cooper‐pair tunneling through precise control of the GdN barrier thickness [87].

Second, fabrication precision is required to suppress the first‐order Josephson coupling. Cooper‐pair tunneling lifts the degeneracy of the logical states, reducing anharmonicity and qubit protection. While a small degree is tolerable and can even help reduce charge‐noise sensitivity, protection and anharmonicity are lost once the first‐ and second‐order Josephson couplings become comparable. Therefore, precise control of the twist angle is important when using a twisted bilayer.

Third, quasiparticle tunneling must be suppressed. Nodal quasiparticles survive in the d‐wave order parameter down to low energies. When quasiparticles tunnel across a junction, they absorb the qubit energy and depolarize the qubit. Fortunately, the energy barrier in the double‐well potential of the d/s junction and the momentum mismatch from twisting in d/d junctions can strongly suppress quasiparticle tunneling [17, 85]. This resembles the gap engineering discussed in Section 3.2.

Integrating d‐wave SCs into superconducting quantum processors presents an exciting and complex challenge for materials science. Despite their potential, d/s SC bilayers remain largely unexplored, with key issues such as lattice matching, interfacial coherence, and compatible growth temperatures requiring careful consideration [17]. For twisted bilayer structures, mechanical exfoliation and transfer are currently the state‐of‐the‐art fabrication methods, but they are inherently handcraft‐intensive, show significant variability across fabrication rounds, and are not scalable at this stage, making them suitable only for proof‐of‐concept demonstrations with single or two qubits. Achieving scalable integration will ultimately depend on developing CMOS‐compatible, wafer‐scale, reliable growth techniques for emerging materials, including high‐quality d‐wave SCs. Prospective strategies are outlined in Subsection 4.3.

#### 3.5.2 π‐Josephson Junctions

π‐Josephson junctions (π‐JJs), characterized by an intrinsic π‐shift in their current‐phase relation (CPR), present a compelling pathway toward scalable, intrinsically protected qubits. In contrast to conventional Cooper pairing, where electrons pair with opposite spins and momenta, a ferromagnetic interlayer can introduce a magnetic exchange energy that lifts spin degeneracy. To compensate, each electron in the Cooper pair acquires a finite additional momentum, resulting in a net center‐of‐mass momentum for the pair. This momentum leads to spatial oscillations of the superconducting order parameter within the ferromagnetic interlayer. Thus, the phase shift arises naturally in systems that break time‐reversal symmetry, such as SC/ferromagnet/SC (SFS) JJs [33, 220, 221, 222]. If inversion symmetry is preserved, the CPR constrains the equilibrium phase difference to either 0 or π, depending on the sign of the effective Josephson coupling. Thus, by precisely tuning the ferromagnetic interlayer thickness, an intrinsic π‐phase shift can be engineered, enabling the realization of π‐junctions with tailored quantum properties.

Since Ryazanov's seminal demonstration of the π‐Josephson effect in a CuNi alloy over two decades ago, a diverse range of π‐JJs has been realized using conventional ferromagnetic materials, alloys, and 2D vdW ferromagnets [220, 223]. However, only a few exhibit properties suitable for quantum computing applications. First, the characteristic decay (or oscillation) length of superconducting pair correlations in ferromagnets, which corresponds to the ferromagnetic interlayer thickness and governs the transition between the 0 and π states, must be longer than ∼1 nm to be experimentally realizable, yet short enough to exhibit a target critical current. Strong ferromagnets, with their large exchange splitting, suppress superconducting coherence, making them unsuitable. Second, the magnetic barrier must be both uniform and magnetically soft. For instance, while Ni can carry supercurrent effectively, its magnetic hardness requires large initialization fields and can lead to domain formation, limiting junction size and critical current. Third, dissipation must be minimized by ensuring the Stewart–McCumber parameter, $\beta_{c}=\left(2e/\hbar \right)I_{c}R_{N}^{2}C$, is larger than 1, which corresponds to an underdamped regime. In superconducting circuits incorporating SFS junctions as passive phase shifters, the Caldeira–Leggett model predicts that loss is minimized when the junction is underdamped [224]. This may help explain the limited coherence (∼1 µs) observed in superconducting qubits integrating overdamped SFS junctions [225]. Therefore, for practical quantum applications, the ferromagnetic barrier must be underdamped, magnetically uniform, and capable of sustaining a sufficiently large critical current density.

Ferromagnetic insulators are especially promising for π‐JJs because their insulating character can suppress quasiparticle transport while still enabling a π‐shifted Josephson coupling [226]. In particular, 2D vdW ferromagnets provide atomically sharp interfaces and precise layer‐by‐layer control [18, 88, 89]. π‐JJs based on 2D vdW materials have been demonstrated using ${\mathrm{NbSe}}_{2}$/${\mathrm{Cr}}_{2}{\mathrm{Ge}}_{2}{\mathrm{Te}}_{6}$/${\mathrm{NbSe}}_{2}$ heterostructures, where ${\mathrm{NbSe}}_{2}$ serves as the superconducting electrode of choice due to its compatibility with 2D vdW materials and its resilience to high in‐plane magnetic fields (30–40 T) without flux trapping. A clear 0‐π transition is observed at a barrier thickness of 8.4 nm, corresponding to a critical current density of 40 $\mathrm{nA}/\mu m^{2}$ [89]. However, this low critical current density underscores the need for alternative ferromagnetic‐insulator‐based π‐junctions. Promising candidates include sputtered NbN/GdN/NbN junctions, which exhibit 0‐π transitions. More broadly, recently explored 2D vdW ferromagnetic metals such as ${\mathrm{Fe}}_{3}{\mathrm{GeTe}}_{2}$ may provide additional routes toward engineered π‐junction behavior, although their suitability as low‐loss barriers remains unexplored [227, 228]. Below, we elaborate on strategies for implementing superconducting qubits based on π‐JJs.

Before full integration in a complex circuit, preliminary experiments should validate the performance of a candidate π‐JJ. One approach is to shunt the π‐JJ with large superconducting electrodes, forming a transmon‐like circuit. This enables the extraction of its Josephson energy, which can be compared with current–voltage measurements and optimized by adjusting the junction size and barrier width. High‐throughput characterization is possible by fabricating multiple junctions with varying dimensions on a single chip. Once optimal parameters are identified, the π‐JJ can be placed in parallel with a 0‐JJ to form a SQUID—both confirming its intrinsic phase shift and benchmarking the symmetry between the zero and π junctions.

We propose two conceptually distinct architectures. The first uses a π‐JJ as a passive phase shifter within a circuit‐engineered effective CQT element, whereas the second aims to exploit a junction whose intrinsic CPR is already dominated by the second harmonic. In the first approach, shown in Figure 8(b), a superconducting circuit can be designed in a rhombus geometry consisting of a 0‐JJ (such as a tunnel junction), a π‐JJ, and superconducting wires. This setupdeep ultraviolet (DUV) photolithography resembles the effective CQT junction realized with Al/${\mathrm{AlO}}_{x}$/Al junctions, where the superinductors enable higher‐order Cooper‐pair tunneling [83, 215]. However, a π‐JJ eliminates the need for external magnetic‐flux control, reducing control‐line noise and improving scalability by decreasing the number of control parameters. A key challenge in this architecture is ensuring that the Josephson energies of the 0‐ and π‐JJs are precisely matched, as any asymmetry can reintroduce Cooper‐pair tunneling, thereby weakening parity protection. In the second approach, shown in Figure 8c, symmetry constraints can be circumvented by directly leveraging the intrinsic CQT of the π‐JJ, providing a conceptually simpler route toward parity‐protected qubits. Second‐harmonic contributions in the CPR have been observed in NbN/GdN/NbN junctions, suggesting a promising route toward achieving CQT‐dominated behavior [87]. Near the precise 0‐π transition point, the first‐harmonic Josephson term can be strongly suppressed, allowing the second harmonic to dominate. Additionally, shunting the junction with a large capacitor can provide parity protection, similar to the d‐wave qubit discussed earlier. Achieving this goal will require precise control of the barrier thickness and a sufficiently large CQT amplitude. Efforts in this direction will pave the way for π‐JJ‐based qubits to become a viable platform for robust, scalable quantum computation, as the required nonlinearity can emerge directly from junction physics rather than from circuit elements, providing a promising route toward materials‐enabled protected superconducting qubits.

### 3.6 State of Play of Material Platforms

Thus far, we have introduced a range of material platforms that aim to advance superconducting quantum processors by improving coherence, tunability, scalability, and intrinsic protection. Here, we assess the current state of these approaches, highlighting their relative maturity, remaining challenges, and prospects for future development.

Qubit relaxation and coherence times, $T_{1}$ and $T_{2}$, together with their corresponding frequency‐normalized quality factors, remain central benchmarks of material and circuit performance, although gate fidelity, leakage, frequency stability, and fabrication yield are also essential for assessing processor‐level relevance. To date, the best reported qubits incorporating SC/SM JJs (Section 3.3.1) or vdW JJs (Sections 3.3.2 and 3.4) generally exhibit coherence times at least an order of magnitude, and often two or more orders of magnitude, below those of state‐of‐the‐art conventional transmons [16, 74, 146]. However, these devices largely represent proof‐of‐principle demonstrations rather than optimized implementations. For epitaxial Al/InAs gatemon circuits, reported limitations include dielectric and substrate loss, microwave loss from the III–V heterostructure and superconducting films, and additional contributions from the electrostatic gate stack, rather than an established intrinsic coherence limit of the semiconductor weak link itself [16, 71]. For graphene‐based gatemons, the available measurements have not yet isolated a single intrinsic junction‐limited decoherence mechanism; radiative coupling through the gate and readout environment, dielectric participation, charge‐noise‐induced frequency fluctuations, and fabrication‐dependent interface effects must still be disentangled [74]. Continued improvements to the surrounding materials and device architecture will therefore be necessary before the intrinsic quality of these junctions can be fully realized.

More broadly, the present performance gap also emphasizes that improvements in an individual material or junction metric do not necessarily translate into improved overall qubit performance. Atomically ordered interfaces may reduce certain disorder‐related contributions to TLS loss. Still, additional decoherence channels can arise from transfer‐induced contamination, edge and interface defects, dielectric loss, charge and critical‐current fluctuations, subgap‐state occupation, and quasiparticle poisoning. Likewise, a high‐κ material does not intrinsically produce greater dielectric loss; however, compact high‐κ or merged‐element capacitor–junction architectures may concentrate electric fields in the intentionally introduced dielectric and its interfaces, increasing their energy participation and thereby enhancing relaxation when their loss tangents are insufficiently low. Gate tunability provides convenient frequency control but can introduce sensitivity to gate‐voltage and charge fluctuations in proportion to the operating‐point‐dependent dispersion $\partial \omega_{q}/\partial V_{g}$; operation near gate‐voltage sweet spots and careful filtering and grounding can partially mitigate this sensitivity. Therefore, crystallinity, compactness, and tunability should be regarded as potential advantages that require validation under circuit‐relevant microwave conditions [229, 230, 231, 232, 233]. Candidate materials and junction architectures must first be evaluated at millikelvin temperatures and in the single‐photon regime using geometry‐controlled resonator loss and participation measurements, followed by direct measurements of qubit relaxation, Ramsey and echo dephasing, frequency stability, anharmonicity, leakage, and single‐ and two‐qubit gate fidelities.

In relative terms, SC/SM JJs are presently more mature than vdW JJs in fabrication yield and reproducibility, although neither platform has yet approached the wafer‐scale uniformity and process maturity of conventional Al/AlOx/Al junctions. Sustained academic and industrial efforts toward topological quantum computing have driven the development of epitaxial SC/SM heterostructures, selective‐area growth, and lithographically defined junctions [73, 194, 234, 235]. By contrast, most qubit‐level demonstrations of vdW JJs still rely on manual exfoliation, transfer, and alignment, resulting in flake‐to‐flake variability, contamination from polymer residues or trapped interfacial bubbles, and inconsistent contact transparency. Scalable synthesis, deterministic transfer, and wafer‐level integration techniques are beginning to emerge. However, fabrication of vdW‐based qubits requires expertise in both superconducting circuit fabrication and two‐dimensional materials processing, substantially slowing the development cycle.

Accelerating progress in vdW‐based qubits will likely require compelling demonstrations of capabilities that cannot be readily achieved with conventional junctions. Parity‐protected qubits, discussed in Section 3.5, provide one promising direction. Although complete parity‐protected qubit implementations based on d‐wave superconductors or SFS junctions remain largely at the proposal stage, experimental observations of dominant second‐harmonic or coherent‐quartet‐tunneling‐like Josephson behavior provide partial validation of the underlying circuit element. Similar to graphene gatemons, twisted bilayer BSCCO could potentially be incorporated into superconducting circuits, but realizing an intrinsically protected qubit would additionally require precise control of the twist angle, suppression of the residual first Josephson harmonic, sufficiently large shunt capacitance, and mitigation of dissipation associated with nodal quasiparticles. As discussed in Section 3.5, avoiding an externally flux‐biased interference loop could reduce flux‐control complexity and remove one potential source of dephasing, but it does not by itself guarantee long $T_{1}$ or $T_{2}$ times; charge–flux dephasing trade‐offs, quasiparticle loss, dielectric loss, and control‐induced coupling must still be quantified [236]. Combined with emerging advances in scalable vdW fabrication techniques (Section 4.3), such demonstrations could catalyze broader efforts toward vdW‐based quantum processors.

Looking forward, we envision two primary routes by which new material platforms can achieve widespread adoption: compatibility with existing superconducting circuit architectures or the demonstration of compelling advantages over conventional Al/AlOx/Al junctions. Because the transmon has become the dominant qubit platform in both academia and industry, many enabling technologies, including foundry‐compatible fabrication (Section 4.2), flip‐chip integration, and through‐silicon vias, have been developed around established transmon processes. Gatemon‐based couplers (Section 3.3) are functionally compatible with this framework because they can provide electrically controlled coupling without requiring a local magnetic‐flux‐bias loop. By separating the coupler and qubit onto different chips using three‐dimensional integration, each component can be fabricated using an optimized process without requiring full materials compatibility. A recent demonstration of vertical tunable couplers based on conventional Al/AlOx/Al junctions in a multilayer transmon architecture illustrates the feasibility of this component‐separated approach [237]. A remaining challenge, however, is ensuring that the additional chip, bonding interfaces, wiring structures, and coupler electrodes do not increase substrate, surface, or interfacial dielectric participation sufficiently to degrade qubit coherence; recent simulations indicate that increased connectivity can measurably enhance such loss channels [238].

Alternatively, emerging material platforms must provide clear performance advantages to justify the additional fabrication complexity. The rapid follow‐up and broader adoption of process‐compatible material modifications, as illustrated by tantalum electrodes and superconducting‐gap‐engineering strategies for transmons, show how clear coherence or robustness benefits can drive adoption [63, 146]. For protected qubits, coherence times comparable to those of state‐of‐the‐art transmons would be highly compelling, but raw $T_{1}$ and $T_{2}$ values alone are not sufficient benchmarks; protected‐state lifetime, noise bias, leakage, gate and readout fidelity, calibration complexity, and the resulting logical error rate and error‐correction overhead must also be evaluated. Miniaturized qubits must meaningfully improve device density and reduce routing area without sacrificing coherence, anharmonicity, controllability, or fabrication yield; a smaller qubit footprint alone does not resolve the cryogenic‐wiring bottleneck, which will also require multiplexing, three‐dimensional integration, and scalable cryogenic control electronics. Equally important, these advances must extend beyond isolated qubits to include high‐fidelity two‐qubit gates, scalable connectivity, and practical control schemes. An experimental example is provided by the surface‐code error budget in Appendix D, which separates contributions from gate operations, data‐qubit idling, readout, and reset. Protected qubits often require specialized protocols to initialize, manipulate, couple, and read out encoded states without breaking protection, whereas miniaturized qubits may require inductive, Josephson, or actively tunable couplers because reduced electrode dimensions weaken direct capacitive interactions. Despite these challenges, research on novel material platforms continues to expand across both academia and industry, suggesting a promising future in which materials innovation plays an increasingly central role in the advancement of superconducting quantum computing.

## 4 Challenges and Opportunities in Nanofabrication

The remarkable progress of the CMOS IC chip industry has been driven by coordinated advances in lithography, deposition, etching, process integration, and packaging [239, 240, 241]. Superconducting quantum technologies will likewise require a multidisciplinary nanofabrication strategy, but with an added emphasis on ultralow‐loss materials, pristine interfaces, and cryogenic reliability.

A key fabrication challenge is precisely depositing superconducting thin films with minimal defect density while maintaining high interface quality. Josephson junction patterning and device architecture optimization must also ensure low‐loss interfaces and reproducible junction characteristics. Moreover, as quantum devices operate in cryogenic environments, ensuring long‐term reliability under extreme conditions is essential. Addressing these challenges is crucial to achieving scalable, high‐performance, industrially viable quantum devices.

This section examines the state‐of‐the‐art fabrication techniques critical to JJ‐based quantum technologies and explores their scalability and integration potential. Multi‐angle evaporation, a widely used method in superconducting circuit fabrication, is analyzed in Subsection 4.1, where we discuss its role in JJ formation, its impact on junction uniformity, and potential improvements. Subsection 4.2 explores the transition from university‐scale fabrication to foundry‐compatible CMOS processes, addressing key process adaptations required to bridge the gap between conventional CMOS IC chip manufacturing and quantum circuit fabrication. In Subsection 4.3, we introduce the integration of novel materials via mechanical exfoliation for proof‐of‐concept and discuss directions for interface control and large‐scale material synthesis.

### 4.1 Conventional Multi‐Angle Evaporation for JJ

Since the 1970s, researchers have fabricated Josephson tunneling junctions by multi‐angle evaporation of superconducting electrodes with an insulating layer between them [242]. Recent decades have seen remarkable advancements in this method, accelerating the development of superconducting quantum computing technologies. These advances have been driven by dramatic improvements in material quality and interfaces, alongside enhanced yields enabled by robust lithography techniques. Building on these developments, many academic and industrial groups have adopted a three‐stage manufacturing procedure that now routinely achieves coherence times in the hundreds of microseconds [45, 46, 243].

Photolithography is the first stage of the manufacturing process and is used to fabricate the quantum processor's linear microwave components. This begins with thoroughly treating the substrate to ensure a pristine interface that minimizes TLS. Common substrate choices include sapphire, valued for its low‐loss properties but challenging to process because of its hardness, and high‐resistivity silicon, which benefits from a well‐established industry but suffers from higher surface and bulk losses. The first treatment is a Piranha solution (a mixture of sulfuric acid and hydrogen peroxide) to remove organic residue [146]. Additionally, for Si substrates, a mixture of ammonium fluoride and hydrogen fluoride, i.e., buffered oxide etchant (BOE), is used to remove lossy surface ${\mathrm{SiO}}_{2}$ [244]. Minimizing the transfer time from substrate cleaning to the deposition chamber is also essential to prevent recontamination and ambient oxide regrowth [153].

After cleaning, the SC is deposited onto the substrate. Metals such as Al and Nb can be sputtered directly. On the other hand, Ta, which has recently been favored for its low‐loss surface oxide, requires either heating the substrate to $∼500^{\circ }C$ or depositing a thin Nb seed layer to promote the growth of the α‐Ta phase, which offers a superior critical temperature and lower loss compared to β‐Ta [146, 245]. Following deposition, photoresist is spin‐coated, and microwave components are patterned using a near‐ultraviolet laser. With a resolution of ∼1μm, this method suits linear components (>10μm) and offers a fast write time (≲ 100 min for a 300 mm wafer) compared to slower, high‐precision techniques like electron‐beam (e‐beam) lithography. Finally, the exposed resist is developed, and the unwanted metal is etched away, revealing the defined processor components.

In the second stage, e‐beam lithography is used to fabricate the JJs. Before Al deposition, the substrate's ambient oxides must be removed. This can be achieved using BOE for Nb and Ta. For Al, this is not possible since Al is etched by BOE [137, 148]. Alternatively, ion milling in the junction‐deposition chamber can be used instead of wet etching. However, special care must be taken to avoid excessive surface roughness due to the bombardment of ions [246, 247, 248].

After treatment, the junctions are patterned onto a spin‐coated e‐beam resist bilayer, enabling the formation of bridges and overhangs required for shadow evaporation. This step is crucial for meeting the yield and uniformity requirements outlined in Subsection 3.1. There are two main techniques for defining junctions. The Dolan style uses a suspended resist bridge to shadow the evaporated aluminum, with junction dimensions determined by the bridge width/length, evaporation angle, and resist height [242]. This method, essential for high‐inductance superconducting circuits, is sensitive to resist‐height variations [11]. In contrast, the Manhattan style relies on a cross‐shaped trench that precisely defines the junction size, making it resilient to resist variations [249]. However, the orthogonal geometry complicates fabricating junction arrays. The choice of evaporation technique ultimately depends on the specific superconducting circuit and application.

Regardless of the lithographic style, a uniform, defect‐free resist coating is essential for accurately defining the junctions. This requires a consistent ambient pressure during spin coating to avoid defects [45]. For e‐beam lithography, proximity effect correction is applied to account for electron backscattering. Nonconductive substrates, such as sapphire, require additional care to prevent charge buildup, which can be mitigated by depositing a thin metal layer over the coated resist [146, 250, 251]. Finally, the developer's temperature, duration, and sonication must be carefully optimized to avoid underdevelopment or overdevelopment and to preserve the integrity of overhanging patterns [45, 46, 243].

The junctions are finalized using double‐angle evaporation of Al, with in situ oxidation between the depositions. Al is the electrode of choice for evaporation because it has a relatively low melting point compared to Nb or Ta, which require sputtering. Furthermore, the thin, self‐limiting Al oxide is a suitable tunnel barrier, as its thickness determines the Josephson energy. The oxidation pressure and duration must be carefully calibrated to achieve the desired qubit parameters. Dynamic oxidation, which maintains constant pressure by modulating oxygen flow, offers better reproducibility and uniformity than static oxidation, which introduces a fixed initial amount of oxygen [45, 50, 51, 120]. However, some nonuniformity is unavoidable because the sample is at a finite distance from the Al source in the evaporation chamber, causing radial variations in the evaporation angle [45, 120]. To mitigate this, the junction dimensions can be pre‐adjusted to compensate for radial variability.

In addition to the oxidation and lithographic parameters, the thickness of the deposited electrodes plays a critical role in qubit performance. By varying the junction electrode thickness, quasiparticle tunneling across the junction can be suppressed (Subsection 3.2). In superconducting thin films, the film thickness directly influences the superconducting gap. By engineering a gap difference larger than the qubit energy, quasiparticle tunneling, and thus qubit decay, can effectively be suppressed. For example, a gap difference exceeding h×10GHz, larger than typical qubit frequencies, can be achieved with a bottom electrode thickness of 10nm and a top electrode thickness of 100 nm. After depositing the top electrode, the junctions are defined by lifting off the resist and excess metal using a solvent.

In the final stage, a patch layer is deposited to establish a reliable electrical contact between the first two stages [59, 243, 252]. The lithography process mirrors the second stage, but without any prior surface treatments. The patches are patterned at the intersections of the base layer and junction electrodes. Ion‐milling removes ambient oxides in the patch region, followed by Al evaporation to form a strong electrical connection. After lift‐off, the fabrication of the superconducting quantum processor is complete. The complete assembly, containing dozens of processor dies, can be probed at room temperature to sieve for defects. Those that meet operating standards are packaged into a printed circuit board box and loaded into the cryostat.

The three‐stage process can be adapted to improve processor scalability. Greater scalability requires transitioning from planar geometries to out‐of‐plane interconnects [253]. Multi‐chip architectures separate quantum components from control components, placing them on two distinct chips connected via flip‐chip bump bonding. An optional third chip, featuring metalized TSVs, can be placed between the chips to enhance isolation [246, 254]. The control chip is typically fabricated using photolithography, while the quantum chip follows a three‐stage process. Flip‐chip bump bonding requires specialized tools and techniques to deposit indium pillars and precisely align the chips [255]. Although this adds complexity, it offers significant advantages, including efficient signal routing, reduced crosstalk, and independent optimization of surface treatment and passivation [60, 256, 257, 258]. Alternatively, out‐of‐plane interconnections can be achieved using spring‐loaded pogo pins, machined structures, or adhesives [259, 260, 261, 262, 263], providing a versatile pathway to scalability.

Finally, while shadow‐evaporated JJs have advanced remarkably, inherent limitations may hinder the progress of superconducting quantum technologies. The primary challenge is that metal deposition occurs after resist patterning, leading to three key issues. First, the pristine substrate‐metal interface is disrupted (or undone) by air exposure during lithography. Second, materials that require high‐temperature deposition are incompatible since heat destabilizes and deforms organic resist. Third, dangling resist polymers can contaminate the electrodes and the junction barrier, contributing to junction aging and performance degradation over time. In addition to these issues, angled evaporation is sensitive to resist height, which reduces junction uniformity. These challenges have spurred growing interest in etch‐based fabrication of JJs, which will be explored in Subsection 4.2.

### 4.2 Toward Superconducting Quantum Foundries

As quantum technology evolves beyond its early exploratory phase toward scalable, high‐quality devices, transitioning from bespoke academic cleanroom processes to foundry‐compatible, wafer‐scale fabrication will be essential. This transition marks a pivotal shift in quantum device manufacturing, mirroring the SM IC industry's trajectory from academic experimentation to industrial‐scale production of silicon microchips with nanoscale features.

To date, the fabrication of JJ‐based quantum devices has primarily occurred in academic settings, emphasizing rapid turnaround and iterative development using processes tailored to specific experiments. In contrast, SM foundries rely on highly standardized, high‐throughput workflows to produce chips with ultra‐fine (∼10 nm) CMOS transistors at high densities (up to ∼100 billion devices per chip). A comparison of representative process paradigms is provided in Table 2. As the superconducting quantum community begins to adopt the high‐precision infrastructure of conventional silicon foundries, the central challenge is to develop process flows that maintain ultra‐low‐loss materials and interfaces, which are crucial for high‐coherence devices while scaling to industrial levels of reproducibility and yield.

**TABLE 2:** Comparison of prototype‐oriented fabrication and foundry‐compatible manufacturing for JJs, qubits, and superconducting circuits.

Category	Prototype‐oriented fabrication	Foundry‐compatible manufacturing
Primary objective	Rapid iteration, flexible prototyping, and materials exploration	Wafer‐scale uniformity, reproducibility, yield, and process integration
Typical lithography	Electron‐beam lithography (EBL) for junctions; optical lithography or direct‐write lithography for larger circuit features	Deep ultraviolet (DUV) photolithography or other stepper/scanner‐based lithography for wafer‐scale patterning; EBL used mainly for development or niche features
Junction definition	Shadow evaporation with Dolan/Manhattan resist structures; lift‐off‐based Josephson junction (JJ) formation	Subtractive or semi‐subtractive processes, including tri‐layer and layer‐by‐layer JJ flows
Tunnel barrier formation	In situ oxidation during double‐angle evaporation; barrier thickness set by oxidation time/pressure	Predeposited tri‐layer barriers or layer‐by‐layer oxide growth after controlled surface cleaning and oxidation
Interlayer cleaning and oxide removal	Wet etch or in situ ion milling immediately before deposition	Integrated surface preparation modules with controlled native‐oxide removal and tighter process monitoring
Metallization	Predominantly evaporation for Al‐based JJs; sputtering used for base films and some superconducting layers	Sputtering, atomic layer deposition (ALD), physical vapor deposition (PVD), and other wafer‐compatible thin‐film tools with tighter thickness and across‐wafer uniformity control
Pattern transfer	Lift‐off is common; etching used selectively	Dry etching, planarization, spacer‐defined isolation, and additive/subtractive routing designed for scalable integration
Dielectric isolation and planarization	Often minimized or avoided to reduce dielectric loss; limited use of temporary process dielectrics	More extensive use of spacers, planarization, and temporary isolation layers, which must be engineered to avoid added loss
Metrology and process control	Device‐level validation, room‐temperature screening, and iterative tuning of local process conditions	Wafer‐scale process control, inline metrology, across‐wafer critical‐current uniformity, defect monitoring, and statistical yield analysis
Main strength	Fast development cycle and compatibility with unconventional device concepts and novel materials	High reproducibility, tighter parameter spread, backend compatibility, and scalable manufacturing
Main limitation	Limited throughput, weaker wafer‐scale uniformity, and reduced compatibility with industrial integration	Higher process complexity and stricter requirements for loss control, materials compatibility, and integration of nonstandard junction materials

One of the most significant challenges in this transition lies in fabricating high‐quality JJs, which differ markedly from standard SM foundry processes. To achieve a typical JJ feature size of ∼100 nm, researchers have traditionally used electron‐beam lithography (EBL) because it can define features as small as ∼7 nm. EBL is maskless, offers flexibility, and enables rapid iteration, making it well‐suited for prototyping and exploring new device architectures. However, EBL is inherently time‐consuming, prone to errors such as charging effects and electron scattering, and, crucially, not fundamentally required for JJ fabrication at the ∼100 nm scale when deep ultraviolet (DUV) photolithography compatible subtractive flows are available.

Notably, the required feature sizes for JJs are within the resolution limits of DUV photolithography. Consequently, there has been growing interest in adapting foundry‐grade DUV tools for JJ patterning. Emerging approaches generally fall into two categories: (1) tri‐layer junction fabrication on wafers with a uniform junction material stack [121, 124], and (2) layer‐by‐layer patterning strategies incorporating in situ native oxide removal and controlled oxide growth [125, 126]. We examine both approaches in detail, highlighting their advantages and limitations.

#### 4.2.1 Tri‐Layer Process

In the first approach, as shown in Figure 9a, all superconducting and insulating layers forming the JJ are deposited before any lithographic steps. These materials can be grown with atomic‐level precision in ultra‐high vacuum using advanced thin‐film deposition techniques. Because the full tri‐layer stack is deposited before lithography, barrier thickness and electrode geometry can be controlled more uniformly across the wafer. This improves the reproducibility of the junction's critical current density, even if the tunnel barrier remains amorphous, depending on the chosen material system. Most importantly, it suppresses variations in barrier thickness, significantly improving JJ uniformity and consistency at the wafer scale, since a JJ's critical current density depends exponentially on its barrier‐layer thickness. DUV lithography and anisotropic dry metal etching enable the fabrication of JJs with high geometric‐area precision. This subtractive process further improves junction uniformity and consistency across a wafer compared with lift‐off processes, since a JJ's critical current is proportional to its area.

**FIGURE 9:**
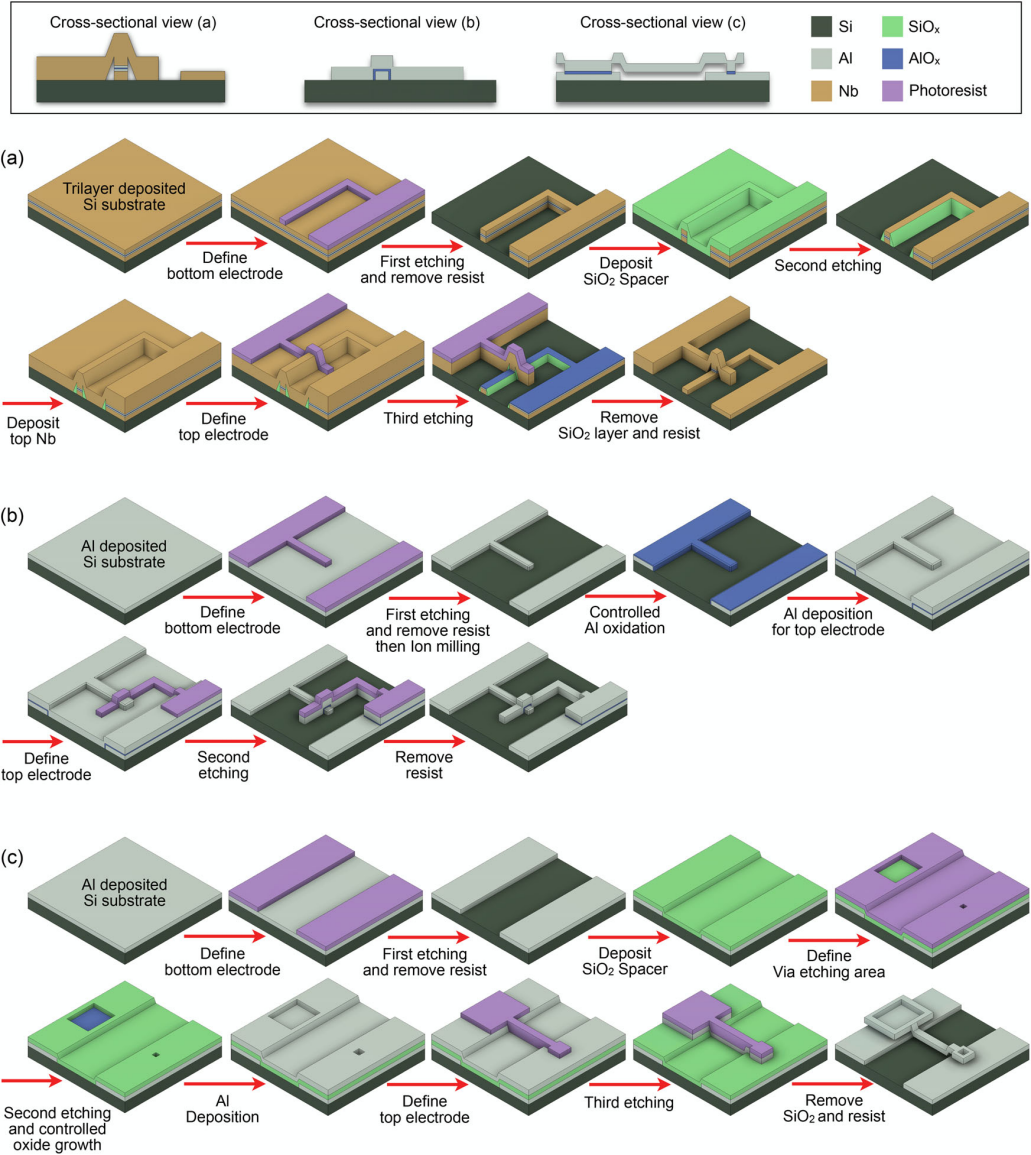
Foundry‐compatible Josephson junction (JJ) fabrication processes at the wafer scale. (a) Nb/Al‐${\mathrm{AlO}}_{x}$/Nb tri‐layer junctions. Multilayers are deposited and patterned using ${\mathrm{SiO}}_{2}$ spacers to define junctions and avoid shorts, followed by top Nb wiring and spacer removal [124]. (b) Al/${\mathrm{AlO}}_{x}$/Al JJs defined by etching. The bottom Al is patterned, ion‐milled, oxidized, and topped with a second Al layer to form the junction [126]. (c) Planarized Al/${\mathrm{AlO}}_{x}$/Al JJs. The bottom Al and circuits are patterned first. Then the ${\mathrm{SiO}}_{2}$ spacer forms via windows and controlled oxidation creates the barrier before the final top Al deposition and spacer removal [125].

While deposition, DUV lithography, and anisotropic etching enable the fabrication of JJs with exceptional uniformity and wafer‐scale consistency, a critical challenge lies in routing current between the bottom and top electrodes without introducing electrical shorts. This step is essential to integrate the junctions into the broader quantum circuit and achieve reliable junction yield across the wafer. Early tri‐layer fabrication approaches [121, 264] addressed this issue by permanently encapsulating the exposed superconducting leads with a lossy dielectric isolation layer. However, this strategy increased dielectric loss, resulting in significantly lower coherence than Al‐based junctions fabricated via angled evaporation.

A recent advancement overcame this limitation by introducing a removable isolation layer [124]. In this approach, a ${\mathrm{SiO}}_{2}$ layer is temporarily deposited on the exposed surfaces of the junction electrodes through an additional deposition sequence, lithography, and etching. This layer provides physical isolation, preventing electrical contact between the existing bottom electrode and the subsequently deposited top wiring layer. Crucially, the isolation material can later be selectively removed, mitigating additional dielectric loss while preserving reliable junction connectivity and offering a scalable, low‐loss solution compatible with advanced quantum circuit architectures.

A critical step in the process outlined in Figure 9a involves the selective removal of the temporary ${\mathrm{SiO}}_{2}$ isolation layer—a delicate procedure requiring careful experimental optimization. Because the ${\mathrm{SiO}}_{2}$ spacer resides beneath the metal wiring, conventional top‐down anisotropic physical etching proves ineffective. Conversely, aggressive isotropic chemical etching risks damaging the underlying junction structure if not correctly optimized for material selectivity. Incomplete removal can also degrade coherence due to residual dielectric loss associated with the amorphous ${\mathrm{SiO}}_{2}$.

Recent work on Nb/Al‐${\mathrm{AlO}}_{x}$/Nb tri‐layer junctions with a temporary ${\mathrm{SiO}}_{2}$ spacer demonstrated improved coherence times approaching 60μs using an optimized selective wet chemical etching protocol [124]. However, finite selectivity and surface tension effects limit this wet etching process, particularly when removing ${\mathrm{SiO}}_{2}$ from confined geometries. Highly selective vapor‐phase hydrofluoric acid etching offers a promising alternative, enabling conformal ${\mathrm{SiO}}_{2}$ removal with reduced risk to the surrounding superconducting structures.

The overall fabrication process proceeds as follows: SCs and insulators are first deposited on a silicon wafer, followed by photolithographic patterning and etching to define the junction electrodes. Then, a ${\mathrm{SiO}}_{2}$ spacer is added and patterned to isolate the bottom and top electrodes during subsequent electrical processing. The top Nb wiring layer is deposited and etched to connect the JJ's upper electrode to the planar circuit. Finally, the ${\mathrm{SiO}}_{2}$ spacer is selectively removed, completing the junction without introducing lossy dielectric interfaces. With further refinement, this process holds promise for scalable, high‐coherence quantum device fabrication compatible with commercial foundry technologies.

#### 4.2.2 Layer‐by‐Layer Process

Alternatively, layer‐by‐layer fabrication approaches define each junction layer through independent lithography steps [125, 126, 265], thereby eliminating the need to deposit and later remove lossy isolation dielectrics. Compared to the traditional tri‐layer method, this strategy offers a cleaner integration route that may reduce contamination pathways and, if oxide removal and surface damage are carefully controlled, improve coherence times. While conceptually similar to the evaporation‐based techniques described in Subsection 4.1, the key distinction lies in directional thin‐film deposition: the junction electrodes are patterned without angled evaporation. Recent work has demonstrated that transmon qubits can be fabricated entirely using foundry‐compatible processes, including thin‐film deposition and subtractive etching, to define both the top and bottom junction electrodes [125, 126], as illustrated in Figure 9b,c.

These layer‐by‐layer methods offer several key advantages. First, depositing the bottom metal layer before lithography enables ultra‐high‐vacuum thin‐film deposition while preserving substrate surface treatments. Second, anisotropic etching of the junction area yields well‐defined sidewalls, improving line‐edge roughness and minimizing radial variability across the wafer. Third, the tunnel barrier forms without organic resists, reducing contamination and improving junction reliability.

As illustrated in Figure 9b, all‐aluminum junction fabrication involves depositing and patterning the Al film to define the bottom electrodes. After ion milling and oxidation to form the ${\mathrm{AlO}}_{x}$ barrier, we deposit and pattern a second Al layer to form the top electrodes. Figure 9c shows that the Al JJ bottom electrode and circuit layer are deposited and patterned first. The ${\mathrm{SiO}}_{2}$ spacer is deposited and patterned to form vias that expose the windows for the JJ and contact areas. The exposed Al bottom layer in the windows is ion‐milled and controllably oxidized to form an ${\mathrm{AlO}}_{x}$ junction barrier layer, followed by the deposition and patterning of the top Al electrode, which forms the junction. Finally, the ${\mathrm{SiO}}_{2}$ spacer is removed by a vapor HF process.

A primary challenge of the layer‐by‐layer approach is the vacuum break between metal deposition steps. Exposure to ambient conditions can lead to the formation of surface oxides on the base electrode, which must be removed before tunnel barrier formation. This is typically addressed via ion milling, in which Ar‐ion bombardment strips away native oxides. However, precise calibration is critical: excessive milling can damage the underlying metal, introduce TLS, and increase surface roughness, while insufficient milling may leave residual oxides that degrade junction performance.

Recent work has demonstrated that wafer‐scale, layer‐by‐layer processes can produce high‐coherence devices despite this challenge. For example, foundry‐compatible fluxonium qubits fabricated with this method achieved lifetimes >1ms although the demonstrated flow still retained evaporation for the top electrode [265]. Furthermore, the tunnel barrier uniformity achievable through in situ controlled oxidation in these layer‐by‐layer processes can rival angled evaporation techniques. Nevertheless, compared with the precisely engineered tunnel barrier in tri‐layer junctions, this method may show reduced uniformity and reproducibility in critical current density, creating a trade‐off between process flexibility and control over device parameters. Continued refinement and optimization of process flows will be essential for producing superconducting JJ‐based qubits with ultra‐high uniformity and coherence.

### 4.3 Integration of Novel Junction Materials: From Proof‐of‐Concept to Scalable Fabrication

Integrating novel junction materials into scalable superconducting circuits requires a staged fabrication pathway. Early proof‐of‐concept devices often rely on mechanically exfoliated materials or highly controlled epitaxial growth to validate junction physics and interface quality. Long‐term adoption in practical quantum hardware, however, requires a shift to wafer‐compatible deposition, patterning, and integration schemes that preserve ultralow‐loss interfaces while delivering high yield and reproducibility. This progression is particularly important for emerging 2D vdW materials, whose promise for superconducting leads, tunnel barriers, ferromagnetic interlayers, and topological weak links is closely tied to the ability to control buried interfaces and structural disorder over large areas.

Conventional SIS JJs have historically been realized using superconductors such as Al [153, 154, 155], Nb [154], Ta [157], Re [53, 152], NbN [52, 54, 55, 156], NbTiN [269], and $\mathrm{Mg}B_{2}$ [270]. Among the available growth routes, molecular beam epitaxy (MBE) provides exceptional control over film purity, stoichiometry, and interface abruptness, but its cost, throughput, and integration complexity limit its widespread use in large‐scale fabrication. Homoepitaxial growth of single‐crystal superconductors remains challenging, while heteroepitaxy on technologically relevant substrates such as Si(111) [50, 153] introduces additional constraints related to lattice mismatch, thermal budget, and process integration. As a result, the key challenge is not merely identifying better materials, but incorporating them into fabrication flows compatible with scalable superconducting circuit manufacturing.

#### 4.3.1 Conventional Superconductors in CMOS‐Compatible Processes

CMOS‐compatible superconducting process flows on 300 mm wafers increasingly rely on deposition routes such as ALD TaN [157], sputtered Al, and controlled oxidation on high‐resistivity Si substrates [122, 126, 269, 271, 272]. More recently, Kr sputtering enabled α‐Ta growth on Si at temperatures as low as 200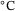, providing a BEOL‐compatible route to high‐performance superconducting circuits [273]. These approaches reduce dependence on MBE while still enabling superconducting films and junctions of useful quality. Their appeal lies in process compatibility, wafer‐scale throughput, and integration with established semiconductor infrastructure. However, trade‐offs remain in film crystallinity, interface abruptness, roughness control, and linewidth precision, all of which can directly influence JJ loss, reproducibility, and critical‐current spread. Continued refinement of deposition chemistry, surface preparation, and interface engineering will therefore be essential to translate conventional superconductors into commercial, foundry‐compatible quantum platforms [52, 54, 55, 156].

#### 4.3.2 Emerging 2D vdW Materials: Current Scalability Limitations

In parallel with conventional superconductors, a broad range of 2D vdW materials is being explored for JJs [14, 16, 18, 74, 75, 78, 88, 89, 177, 204, 274, 275, 276, 277, 278, 279, 280, 281, 282, 283, 284, 285, 286]. Representative families include 2D vdW superconductors such as ${\mathrm{NbSe}}_{2}$ [14, 78, 204, 274, 275] and ${\mathrm{NbS}}_{2}$ [276], semimetals such as graphene [74, 75, 277, 278, 279, 280, 283], topological materials such as ${\mathrm{WTe}}_{2}$ [76, 284, 287] and ${\mathrm{MoTe}}_{2}$ [288, 289], and 2D vdW ferromagnets such as ${\mathrm{Cr}}_{2}{\mathrm{Ge}}_{2}{\mathrm{Te}}_{6}$ [18, 88, 89]. The main bottleneck is no longer proof‐of‐concept device physics, but reproducible synthesis of low‐defect materials and buried interfaces at wafer scale. Although chemical vapor deposition (CVD) enables large‐area growth, grain boundaries, stoichiometric disorder, and high defect densities often degrade low‐temperature electronic and superconducting performance. Chemical vapor transport can yield higher‐quality crystals for laboratory‐scale devices [18, 284, 290], but it alone does not address the challenges of large‐area integration. As a result, 2D vdW materials will likely enter scalable JJ technologies through a staged pathway: (i) proof‐of‐concept validation by exfoliation and transfer, (ii) interface verification and engineering, (iii) direct growth and vertical stacking, and finally (iv) foundry‐compatible patterning and integration.

#### 4.3.3 Exfoliation and Transfer

Mechanical exfoliation should be viewed primarily as a validation platform rather than a manufacturing solution. It has nonetheless played a central role in early‐stage materials screening and device prototyping by enabling researchers to isolate high‐quality, atomically thin flakes from bulk crystals while minimizing grain boundaries and preserving intrinsic superconducting or correlated‐electron properties. This makes exfoliation especially valuable for establishing the relevant figures of merit for candidate JJ materials, including superconducting gap characteristics, interface transparency, transport uniformity, and coherence‐related loss channels.

For JJ fabrication, however, the primary challenge is maintaining clean and chemically stable buried interfaces during transfer and assembly. Interfacial contamination, oxidation, trapped bubbles, strain inhomogeneity, and transfer‐induced damage can all introduce TLS, dielectric loss, and spatially nonuniform transport. Considerable effort has therefore been devoted to interface‐preserving transfer strategies, including in‐vacuum exfoliation [291], h‐BN encapsulation [161, 292, 293], silicon nitride membrane‐assisted exfoliation [294], and inert or trap‐free transfer methods [295, 296]. As summarized in Figure 10, viscoelastic stamping (Figure 10a) offers a simple deterministic transfer route [266]; pick‐up transfer (Figure 10b) is widely used for assembling cleaner buried heterointerfaces; tear‐and‐stack (Figure 10c) enables controlled twist‐angle formation; and patterned transfer (Figure 10d) points toward larger‐area assembly strategies [267]. Machine‐learning‐assisted robotic flake identification and transfer may further accelerate prototyping [297]. Even so, exfoliation‐based JJ fabrication remains fundamentally limited in throughput, reproducibility, and wafer‐scale uniformity, and is therefore best regarded as a proof‐of‐concept route for validating materials and interface concepts before scalable process development.

**FIGURE 10:**
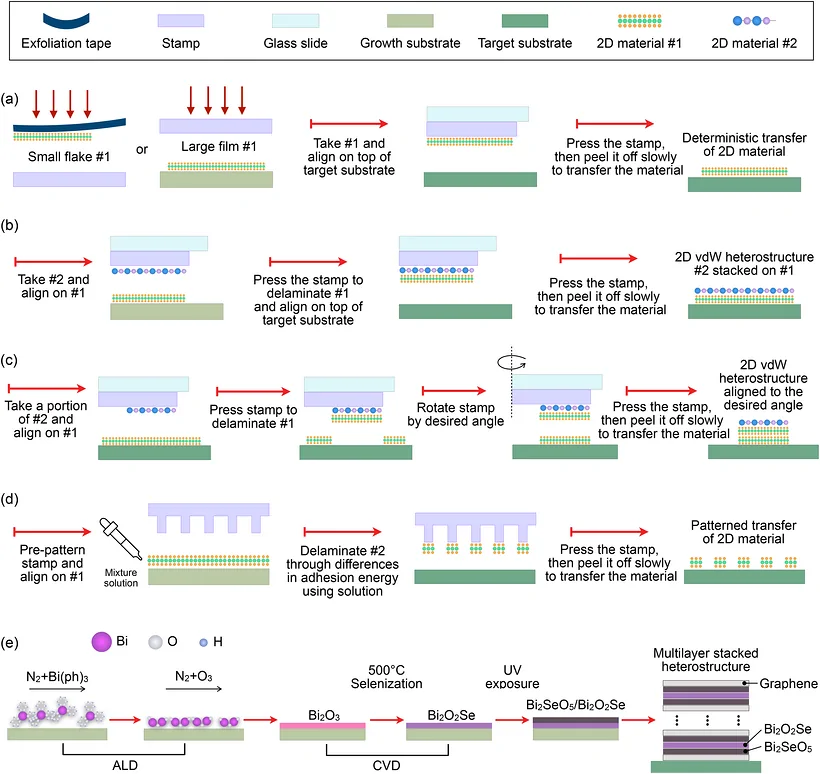
Methods for constructing two‐dimensional van der Waals (2D vdW) materials and their heterostructures. (a) Viscoelastic stamping method for the deterministic transfer of a 2D vdW material [266]. (b) Pick‐up transfer method for assembling a 2D vdW heterostructure. (c) Tear‐and‐stack transfer method for a 2D vdW heterostructure aligned to a desired twist angle. (d) Patterned transfer method for large‐area patterned transfer of 2D vdW materials [267]. (e) Direct growth and stacking method for multilayer 2D vdW heterostructures [268].

#### 4.3.4 Direct Growth and Stacking

Direct growth becomes compelling only when it can simultaneously address material quality, interface abruptness, and process compatibility. For practical integration into foundry‐compatible superconducting circuits, deposition‐based methods must eventually replace exfoliation. MBE remains the benchmark for crystalline control and low‐defect thin films, but its low throughput and demanding UHV requirements limit large‐scale manufacturing. By contrast, ALD offers atomic‐scale thickness control and conformality, particularly attractive for tunnel barriers and interface engineering, while physical vapor deposition (PVD)‐based methods, such as sputtering, are highly compatible with existing superconducting process flows [273]. CVD enables large‐area growth of many 2D vdW materials, although grain boundaries and nonuniform stoichiometry remain major concerns. Recently, hybrid and plasma‐assisted routes have emerged as promising bridges between material quality and process scalability [269, 272, 298]. And more recently, encapsulation epitaxy enabled larger than 1‐inch, air‐stable monolayer ${\mathrm{NbSe}}_{2}$ films and their integration into superconducting circuits, providing a route toward wafer‐scale 2D vdW quantum circuitry [68].

Figure 10e illustrates the broader concept of direct growth and stacking for multilayer 2D vdW heterostructures. In one example, ALD grows ${\mathrm{Bi}}_{2}O_{3}$, which CVD selenizes to form semiconducting ${\mathrm{Bi}}_{2}O_{2}\mathrm{Se}$; UV‐assisted intercalative oxidation then converts the top layers into an epitaxial ${\mathrm{Bi}}_{2}{\mathrm{SeO}}_{5}$ dielectric [268]. Although this example originates from gate stack engineering for logic transistors and does not itself constitute a superconducting junction, it demonstrates a general sequential growth‐and‐conversion strategy through which wafer‐scale, vertically integrated vdW heterostacks with tailored interfaces may be realized. Such an approach is directly relevant to future JJ tunnel barriers and multilayer superconducting stacks. For quantum devices, the corresponding challenge is to achieve comparable control while preserving superconducting properties, minimizing interdiffusion, and suppressing losses from defects, traps, local strain, and imperfect buried interfaces.

More generally, scalable 2D vdW JJ fabrication will require high‐quality superconducting leads, uniform tunnel barriers or gap interlayers, and, where needed, topological or ferromagnetic proximity layers. The relevant interface engineering must extend beyond SC/SC boundaries to include SC/dielectric, SC/substrate, and SC/vacuum interfaces. Reducing TLS‐induced dielectric loss, charge trapping, and local structural disorder is essential for transforming promising 2D vdW junction concepts into reproducible, high‐coherence qubits [23].

Among layered superconductors, ${\mathrm{NbSe}}_{2}$ and ${\mathrm{NbS}}_{2}$ represent promising candidates for directly grown superconducting electrodes because they provide intrinsic superconductivity in the few‐layer regime and are compatible with vapor‐phase or epitaxial growth approaches [299, 300]. Layered ${\mathrm{TaS}}_{2}$‐based materials may offer additional possibilities, although their superconducting behavior can be sensitive to thickness, crystalline phase, and charge‐density‐wave ordering [301]. For an all‐vdW SC/dielectric/SC configuration, atomically thin h‐BN is a natural tunnel‐barrier candidate, leading to prospective structures such as ${\mathrm{NbSe}}_{2}$/h‐BN/${\mathrm{NbSe}}_{2}$ or ${\mathrm{NbS}}_{2}$/h‐BN/${\mathrm{NbS}}_{2}$. Semiconducting TMDs, including ${\mathrm{MoS}}_{2}$ and ${\mathrm{WSe}}_{2}$, may alternatively serve as tunable gap or weak‐link interlayers in SC/semiconductor/SC junctions. From a near‐term process‐integration perspective, hybrid structures combining directly deposited vdW SCs with ultrathin ALD‐grown dielectrics may also provide a practical intermediate route, although nucleation, plasma‐induced damage, barrier defects, and dielectric loss must be carefully controlled. Therefore, the transition from exfoliation‐based all‐2D vdW JJs to deposition‐based, foundry‐compatible processes is not merely a question of throughput, but of establishing a fabrication ecosystem in which material growth, interface control, patterning, and circuit integration are co‐optimized for uniformity, yield, and coherence. Moreover, these material combinations should be regarded as promising integration targets rather than established foundry‐ready JJ platforms.

#### 4.3.5 Post‐Fabrication Tunability through MEMS Integration

Recent advances in MEMS (microelectromechanical systems) provide a complementary route to improve 2D vdW JJ technologies through post‐fabrication trimming and tunability [302, 303, 304]. MEMS actuators enable controlled adjustment of twist angle, interlayer spacing, and mechanical strain, thereby expanding the design space for highly reproducible quantum devices based on 2D vdW heterostructures or d/s superconducting bilayers. Such control is particularly attractive for twist‐angle‐dependent Josephson devices and other twistronic superconducting platforms, where the relevant quantum phenomena can depend sensitively on relative alignment and interfacial spacing [283, 305].

Beyond enabling in situ tunability, MEMS platforms also provide a practical post‐fabrication correction strategy for otherwise unavoidable fabrication errors, including angle misalignment, strain inhomogeneity, and imperfect interfacial contact [304]. Mechanical actuation can additionally suppress wrinkles and trapped bubbles [306], both of which are known to generate charge traps, dielectric loss, and spatially inhomogeneous transport. In this sense, MEMS integration is not only a route toward new device functionalities but also a potentially valuable tool for improving yield, reproducibility, and interface quality in future twist‐angle‐sensitive superconducting quantum circuits.

## 5 Outlook

The Josephson junction (JJ) has served as the fundamental building block of superconducting quantum circuits for more than six decades and remains central to modern quantum technology [2, 3, 28]. What has changed is the scale at which it must now operate. A junction that performs adequately in a two‐qubit experiment may be insufficient in a processor containing thousands of qubits, where fabrication variability, frequency crowding, and device imperfections accumulate across the chip [2, 91]. In such systems, the worst‐performing junction can ultimately limit the entire processor's performance. The central question for the field is therefore no longer whether JJs can support quantum computation, but whether they can do so with the uniformity, stability, and scalability required for fault‐tolerant architectures [1, 91].

In Section 3, we examined five key challenges that will shape the future of JJs in quantum processors: yield and reproducibility, dissipation, tunability, device footprint, and intrinsic noise protection. On the conventional Al/${\mathrm{AlO}}_{x}$/Al platform, sustained engineering progress has driven coherence times beyond one millisecond and junction uniformity to the few‐percent level [10, 146]. These advances have enabled rapid progress in superconducting quantum processors, including multi‐qubit devices and early demonstrations of logical error suppression [2, 90, 91]. At the same time, several limitations are becoming increasingly apparent as systems scale. In particular, the amorphous nature of the ${\mathrm{AlO}}_{x}$ tunnel barrier introduces stochastic TLS that contribute to energy loss and frequency instability [62, 113, 130]. Although extensive process optimization has substantially reduced these effects, their microscopic origins suggest that further gains may ultimately require either alternative barrier materials or new qubit architectures designed to mitigate these loss mechanisms.

Several emerging material platforms offer promising pathways to address these challenges. Crystalline tunnel barriers, SC/SM junction‐based 2DEG systems, 2D vdW materials and their heterostructures, and ferromagnetic interlayers each target different limitations of conventional devices. Crystalline and epitaxial barriers aim to reduce structural disorder and TLS density, while semiconducting materials enable electrostatic tunability that can mitigate frequency collisions and support new circuit functionalities [16, 73]. 2D vdW materials and their heterostructures can provide cleaner interfaces with reduced dangling‐bond disorder than conventional amorphous barriers, while their atomically thin nature also enables device miniaturization [66, 78, 197]. At the same time, unconventional superconducting and magnetic systems enable intrinsically protected qubit architectures based on higher‐order tunneling processes, such as Cooper‐quartet tunneling [17, 85]. Despite these promising directions, most emerging platforms remain at an early stage. Especially, Table 4 shows a lack of reports on RA product dispersion and quantum gate fidelity in emerging material‐based qubit platforms. Therefore, a key goal in emerging material‐based platforms is to investigate gate fidelity and RA product spread through the fabrication strategies featured in Section 4.3. Coherence times and quality factors reported for many emerging junction platforms remain below those of the most mature aluminum‐based qubits [203, 208] (Tables 3 and 4). However, it is worth recalling that aluminum‐based qubits themselves improved by a factor of $10^{6}$ over roughly two decades, from nanoseconds in the early 2000s to milliseconds today, and that much of this improvement arose not from the junction alone, but from advances in substrates, packaging, filtering, and circuit design once the underlying junction platform was established. Unconventional superconducting qubits can have much longer lifetimes [114, 214, 307, 308, 309, 310]. Emerging platforms may follow a similar trajectory, provided material quality, interface control, and scalable fabrication improve in parallel.

**TABLE 3:** Coherence benchmarks across Josephson junction (JJ) material platforms discussed.

Platform	Reported $T_{1}$	Reported $T_{2}$	$T_{2}$ meas	Same device?	Junction Type	Year	Ref.
Al/${\mathrm{AlO}}_{x}$/Al JJ (mergemon)	234 µs	50 µs	Echo	No^a^	Thick tunnel barrier	2021	[207]
Nb/Al‐${\mathrm{AlO}}_{x}$/Nb JJ (trilayer transmon)	62.4 µs	35.4 µs	Echo	Yes	Conventional tunnel barrier	2024	[124]
Al/${\mathrm{AlO}}_{x}$/Al JJ (layer‐by‐layer fluxonium)	1.168 ms	0.943 ms	Echo	Yes	Conventional tunnel barrier	2025	[265]
Al/${\mathrm{AlO}}_{x}$/Al JJ (Ta‐shunted transmon)	1.682 ms	1.185 ms	Echo	No^b^	Conventional tunnel barrier	2025	[10]
Al/InGaAs/InAs/InGaAs/Al JJ (gatemon)	1.1 µs	2.2 µs	Echo	Yes	SC/SM‐based 2DEG systems	2018	[16]
Al/h‐BN/graphene/h‐BN/Al JJ (gatemon)	36 ns	55 ns	Ramsey $T_{2}^{*}$	Yes	2D vdW heterostructure	2019	[74]
Al/${\mathrm{AlO}}_{x}$/Al SQUID (${\mathrm{NbSe}}_{2}$/h‐BN/${\mathrm{NbSe}}_{2}$ shunted transmon)	24.4 µs	25.1 µs	Ramsey $T_{2}^{*}$	Yes	Conventional + 2D vdW	2022	[14]
${\mathrm{NbSe}}_{2}$/${\mathrm{WSe}}_{2}$/${\mathrm{NbSe}}_{2}$ JJ (mergemon)	2.46 µs	4.82 µs	Echo	Yes	2D vdW heterostructure	2025	[208]

^*^ Notes: Echo denotes Hahn/spin‐echo measurements where specified in the cited work. “Same device?” indicates whether the listed $T_{1}$ and $T_{2}$ values were measured on the same qubit.

^a^ For Ref. [207], the listed $T_{1}=234\;\mu s$ and echo $T_{2}=50\;\mu s$ were measured on different devices and do not represent the coherence times of a single qubit.

^b^ For Ref. [10], the best $T_{1}$ and $T_{2}$ values were reported for different qubits (qubits 34 and 17, respectively).

**TABLE 4:** Qubit performance, junction parameters, and reported junction variability across the Josephson junction (JJ) material platforms discussed.

Platform	$Q_{1}$	RA Product Range	$J_{c}$ Range	$I_{c}$ Range	Gate Fidelity	Ref.
Al/${\mathrm{AlO}}_{x}$/Al JJ (mergemon)	$∼1.1\times 10^{6}$ ^med)^	12.7–$14.9k\Omega \cdot \mu m^{2}$ ^b)^	$∼1.9-2.5A\cdot {\mathrm{cm}}^{-2}$ ^i), AB)^	∼24nA ^t)^	NR	[207]
Nb/Al–${\mathrm{AlO}}_{x}$/Nb JJ (trilayer transmon)	$∼2.57\times 10^{5}$ ^m)^	$∼10k\Omega \cdot \mu m^{2}$, ∼5–10% spread	∼1–$20A\cdot {\mathrm{cm}}^{-2}$ ^d)^	NR	NR	[124]
Al/${\mathrm{AlO}}_{x}$/Al JJ (layer‐by‐layer fluxonium)	$∼1.32\times 10^{6}$ ^i), m)^	$∼2.5k\Omega \cdot \mu m^{2}$, <5% spread	$∼11-13A\cdot {\mathrm{cm}}^{-2}$ ^i), AB)^	∼10nA ^i)^	NR	[265]
Al/${\mathrm{AlO}}_{x}$/Al JJ (Ta‐shunted transmon)	$∼9.7\times 10^{6}$ ^m)^	NR	NR	∼15–20nA ^i)^	∼99.994% ^c)^	[10]
Al/InGaAs/InAs/InGaAs/Al JJ (gatemon)	NR^a)^	N/A	NR	∼32–60nA ^i)^	NR	[16]
Al/h‐BN/graphene/h‐BN/Al JJ (gatemon)	$∼1.4\times 10^{3}$ ^i)^	N/A	NR	∼90–360nA ^i)^	NR	[74]
Al/${\mathrm{AlO}}_{x}$/Al SQUID (${\mathrm{NbSe}}_{2}$/h‐BN/${\mathrm{NbSe}}_{2}$ shunted transmon)	$∼4.2\times 10^{4}$–$4.5\times 10^{5}$ ^m), dr)^	NR	NR	NR	NR	[14]
${\mathrm{NbSe}}_{2}$/${\mathrm{WSe}}_{2}$/${\mathrm{NbSe}}_{2}$ JJ (mergemon)	$∼9.0\times 10^{3}$–$6.1\times 10^{4}$ ^i), m), dr)^	NR	NR	∼9–35nA ^i), dr)^	NR	[208]

^*^ Notes: Superscripts indicate the provenance or statistical meaning of the listed quantities: ^m)^ mean, ^med)^ median, ^i)^ inferred, ^dr)^ device range, ^t)^ target, ^d)^ process‐level $J_{c}$ range for PECVD‐processed junctions, and ^AB)^ Ambegaokar–Baratoff estimate. The $J_{c}$ values marked ^AB)^ were estimated from the listed RA products using the zero‐temperature Ambegaokar–Baratoff relation, $J_{c}=\pi \Delta_{\mathrm{Al}}/\left(2eRA\right)$, with an assumed superconducting gap of $\Delta_{\mathrm{Al}}=180-200\;\mu \mathrm{eV}$. These are not device‐specific $J_{c}$ measurements. For quantities marked as inferred, $Q_{1}$ was calculated using $Q_{1}=2\pi f_{01}T_{1}$, and $I_{c}$ was obtained from the reported Josephson energy using $I_{c}=2eE_{J}/\hbar$, where applicable. Values within the same row do not necessarily originate from the same physical device unless explicitly reported as such.

^a^ A qubit $Q_{1}$ was not reported for the Al/InGaAs/InAs/InGaAs/Al gatemon; a test resonator on the same material platform exhibited $Q_{i}≃6.4\times 10^{4}$ at low photon number.

^b^ For Ref. [207], the RA range is inferred from room‐temperature resistance measurements on $2.4\;\mu m^{2}$ test junctions oxidized for 1 h. The coherence data were obtained from qubits fabricated using 4 h oxidation. The corresponding $J_{c}^{\mathrm{AB}}$ estimate uses the room‐temperature resistance as an approximation to the low‐temperature normal‐state resistance.

^c^ The reported fidelity of 99.994% is for single‐qubit gates.

^*^ NR denotes not reported, whereas N/A indicates that the RA product is not directly applicable to the corresponding non‐tunnel‐junction architecture.

Beyond improving superconducting qubit performance, several emerging JJ concepts could expand the functionality of superconducting electronics. Josephson diodes exploit a nonsinusoidal current‐phase relation triggered by broken inversion and time‐reversal symmetry to realize nonreciprocal critical currents, offering low‐dissipation rectification, cryogenic memory, and directional signal routing [5, 282, 311, 312, 313]. Multiterminal JJs extend phase control to three or more superconducting electrodes, where independently tunable phase differences generate engineered Andreev bound states and synthetic topological states such as Weyl singularities [314, 315, 316]. In parallel, researchers are exploring topological JJs based on proximitized topological insulators or spin‐orbit‐coupled semiconductors as hosts or probes for Majorana bound states, with potential relevance to topological quantum computation [317, 318]. More recently, researchers have theoretically investigated altermagnet JJs for crystallographic‐orientation‐dependent 0–π or φ‐junction behavior under vanishing net magnetization, and potentially as a platform for topological superconductivity that reduces the stray‐field and orbital effects associated with conventional SFS JJs [319, 320]. Although these directions remain less mature than conventional Al/${\mathrm{AlO}}_{x}$/Al technology, they have broadened the role of JJs from nonlinear circuit elements to multifunctional platforms for nonreciprocal transport, phase‐coherent routing, and topologically protected quantum computation.

Alongside materials innovation, the fabrication advances discussed in Section 4 will be essential for next‐generation quantum processors. Foundry‐compatible JJ technologies based on optical lithography, trilayer deposition, and etch‐defined junctions have recently demonstrated coherence approaching that of traditional shadow‐evaporated devices, while also offering improved prospects for wafer‐scale uniformity [121, 122]. These developments represent an important step toward industrial‐scale quantum integrated circuits. A useful long‐term analogy is the fabless, foundry model of the semiconductor industry, in which standardized process design rules decouple circuit innovation from manufacturing scale. Superconducting quantum technology has not yet reached this level of maturity, but early signs of a similar ecosystem are emerging. Establishing reproducible junction processes, well‐defined fabrication tolerances, and shared process design rules will therefore be essential to enable scalable quantum hardware development across academic and industrial platforms. Equally important is developing standardized junction metrology, analogous to transistor compact modeling in the semiconductor industry, that systematically links barrier microstructure and transport properties to qubit‐level performance, enabling rapid iteration across fabrication processes and material platforms.

For emerging materials platforms, fabrication presents a different challenge. Many promising demonstrations, particularly in 2D vdW systems, currently rely on mechanical exfoliation and manual assembly [160]. While these approaches are invaluable for proof‐of‐concept experiments and materials validation, they are inherently difficult to scale to large multi‐qubit processors. Bridging this gap will require advances in wafer‐scale materials synthesis, deterministic material placement, and hybrid integration strategies that combine conventional superconducting circuitry with the localized incorporation of new materials only where their unique functionality is required.

Looking ahead, several milestones would mark major inflection points for the field. First, demonstrating qubits based on crystalline materials or 2D vdW JJs with coherence times approaching $T_{1}>100\;\mu s$ would establish that non‐amorphous barriers can support coherence well beyond the proof‐of‐concept regime [10, 146]. Second, realizing a qubit in which higher‐order tunneling processes, such as intrinsic Cooper‐quartet tunneling, dominate the Josephson energy would provide a new route toward hardware‐level noise protection without relying on complex circuit elements [17, 85]. Third, foundry‐fabricated JJs that simultaneously achieve high coherence, tight parameter control, and high yield across large wafers would mark a decisive step toward scalable quantum integrated circuits [121, 126].

The remarkable progress of superconducting quantum processors over the past decade, from few‐qubit experiments to processors containing hundreds or thousands of qubits, has been built on the JJ [2]. As quantum processors continue to scale, the junction itself cannot be treated as a mature, static component. Its materials, fabrication processes, and circuit roles must evolve together with the demands of practical quantum computing. The developments surveyed in this review suggest that the next era of superconducting quantum hardware will be shaped not only by scaling up larger circuits but also by better‐designed, fabricated, and integrated junctions, with the same rigor that transformed semiconductor electronics.

## Conflicts of Interest

The authors declare no conflicts of interest.

## Data Availability

This review article reports no new experimental measurements. The literature data discussed in this review are available in the cited publications. The numerical data and simulation code underlying Figure C2 are available from the corresponding authors upon reasonable request. Figures of merit connecting junction defects and imperfections to physical and logical error rates. (a) Junction barrier two‐level systems (TLS) loss: $T_{1}$ versus loss tangent $\tan\delta_{\mathrm{JJ},\mathrm{eff}}$ for a family of junction field participation ratios $p_{\mathrm{JJ}}$ (Equations C2), at qubit frequency $f_{01}=5\;\mathrm{GHz}$. The $p_{\mathrm{JJ}}=10^{-2}$ and $10^{-3}$ lines represent the typical range corresponding to conventional transmon designs. The $10^{-4}$ line shows the prospective low‐participation bound. (b) The quasiparticle‐limited $T_{1}^{\mathrm{qp}}$ versus normalized quasiparticle (QP) density $x_{\mathrm{qp}}$ (Equation C3). Vertical lines identify values reported in transmon [321, 322] and fluxonium [307, 323]. (c) Surface code logical error per cycle $P_{L}$ versus physical error per operation p for code distances d=3−17 (Equation C6). The shaded band represents the 0.57−1.1% threshold. The star's vertical coordinate is the reported distance‐seven Willow result, while its horizontal coordinate is the effective value $p_{\mathrm{eff}}=p_{\mathrm{th}}/\Lambda$ inferred within this phenomenological model [91]. The right‐hand axes of Figure C1(a) and (b) convert $T_{1}$ into the coherence‐limited average infidelity $p_{\mathrm{coh}}$ for a representative 100 ns operation, assuming $T_{2}=T_{1}$ (Equation C4). Effects from qubit dropout on quantum error correction. Logical failure probability per ideal correction instance, $P_{L}$, as a function of data‐qubit dropout fraction, $f_{\mathrm{TLS}}$. This simplified code‐capacity simulation assumes ideal stabilizer measurements for unrotated planar surface codes. The distances considered are (a) d=3, (b) d=5, and (c) d=7. Missing data qubits are modeled as independently distributed, known erasures, while each surviving data qubit undergoes an independent depolarizing error with probability $p_{\mathrm{op}}=10^{-3}$, $3\times 10^{-3}$, or $10^{-2}$. With ideal stabilizer measurements, the threshold of this model is related to the code capacity, $p_{\mathrm{th}}^{\mathrm{cc}}\approx 15\%$, far above the circuit‐level threshold in Figure C1(c), so the two figures should not be compared at equal error rate. Lines connect the stratified Monte Carlo estimates of Equation (C14) as guides to the eye. Shaded regions show nominal 95% bootstrap intervals obtained by resampling disabled‐site configurations and failure counts, as described in Section C.6. These intervals do not account for possible unobserved failures in strata with zero observed failures and may therefore underestimate uncertainty at the lowest logical failure probabilities.

## Appendix A List of Abbreviations

ABS: Andreev Bound States
ALD: Atomic Layer Deposition
BOE: Buffered Oxide Etchant
CGT: ${\mathrm{Cr}}_{2}{\mathrm{Ge}}_{2}{\mathrm{Te}}_{6}$
CMOS: Complementary Metal‐Oxide‐Semiconductor
CPR: Current‐Phase Relation
CQT: Cooper‐Quartet Tunneling
CVD: Chemical Vapor Deposition
DUV: Deep Ultraviolet
EBL: Electron‐Beam Lithography
EDS: Energy‐Dispersive X‐ray Spectroscopy
EELS: Electron Energy‐Loss Spectroscopy
EOT: Equivalent Oxide Thickness
FET: Field‐Effect Transistor
FinMET: Fin‐shaped Merged‐Element Transmon
FoM: Figure of Merit
IC: Integrated Circuit
JJ: Josephson Junction
MBE: Molecular Beam Epitaxy
MEMS: Microelectromechanical Systems
MET: Merged‐Element Transmon
MWPM: Minimum Weight Perfect Matching
PL: Photoluminescence
PVD: Physical Vapor Deposition
QEC: Quantum Error Correction
QP: Quasiparticle
SC: Superconductor
SM: Semiconductor
SQUID: Superconducting Quantum Interference Device
STEM: Scanning Transmission Electron Microscopy
STM: Scanning Tunneling Microscopy or Standard Tunneling Model
STS: Scanning Tunneling Spectroscopy
TRS: Time‐Reversal Symmetry
TSV: Through‐Silicon Vias
TLS: Two‐Level System
XPS: X‐Ray Photoelectron Spectroscopy
vdW: van der Waals
2D: Two‐Dimensional
2DEG: Two‐Dimensional Electron Gas

## Appendix B Current‐Phase Harmonics

For a short superconducting weak link with transmission eigenvalues $\left\{\tau_{i}\right\}$, the zero‐temperature CPR can be written as

(B1)
$$I\left(\varphi \right)=\sum_{i}\frac{e\Delta }{\hbar }\;\frac{\tau_{i}\sin\varphi }{2\sqrt{1-\tau_{i}\sin^{2}\left(\varphi /2\right)}}.$$

Using the relation sinφ=2sin(φ/2)cos(φ/2), this becomes

(B2)
$$I_{i}\left(\varphi \right)=\frac{e\Delta }{\hbar }\;\tau_{i}\;\sin\frac{\varphi }{2}\cos\frac{\varphi }{2}{\left[1-\tau_{i}\sin^{2}\frac{\varphi }{2}\right]}^{-1/2}.$$

The square root can be expanded using the binomial series with $x=\tau_{i}\sin^{2}\left(\varphi /2\right)$.

(B3)
(1−x)−1/2=∑n=0∞−12n(−x)n=∑n=0∞(2n)!22n(n!)2xn,

Substituting, we obtain

(B4)
$$I_{i}\left(\varphi \right)=\frac{e\Delta }{\hbar }\sum_{n=0}^{\infty }\frac{(2n)!}{2^{2n}{\left(n!\right)}^{2}}\;\tau_{i}^{\;n+1}\sin^{2n+1}\frac{\varphi }{2}\cos\frac{\varphi }{2}.$$

Each term $\sin^{2n+1}\left(\varphi /2\right)\cos\left(\varphi /2\right)$ can be expressed as a finite Fourier sine series, where $a_{n,k}$ are numerical coefficients.

(B5)
$$\sin^{2n+1}\theta \;\cos\theta =\sum_{k=1}^{n+1}a_{n,k}\;\sin\left(2k\theta \right),$$

Setting θ=φ/2 shows that Equation (B4) contains harmonics sin(kφ) with k≤n+1. Therefore, the current admits a Fourier expansion with $I_{k}=\sum_{i}I_{ki}$.

(B6)
$$I_{i}\left(\varphi \right)=\sum_{k=1}^{\infty }I_{ki}\;\sin\left(k\varphi \right),\;I\left(\varphi \right)=\sum_{i}I_{i}\left(\varphi \right)=\sum_{k}I_{k}\sin\left(k\varphi \right),$$

**Small‐Transparency Expansion**

For $\tau_{i}\ll 1$, expanding Equation (B1) to third order yields

(B7)
$$\begin{array}{cc} I_{i}\left(\varphi \right)≃\frac{e\Delta }{\hbar } & \left[\left(\frac{\tau_{i}}{2}+\frac{\tau_{i}^{2}}{8}+\frac{15\tau_{i}^{3}}{256}\right)\sin\varphi -\left(\frac{\tau_{i}^{2}}{16}+\frac{3\tau_{i}^{3}}{64}\right)\sin\left(2\varphi \right)\right.\\ & \left.+\frac{3\tau_{i}^{3}}{256}\sin\left(3\varphi \right)+O\left(\tau_{i}^{4}\right)\right]. \end{array}$$

This explicitly demonstrates that higher harmonics scale as $I_{ki}\propto \tau_{i}^{k}$ in the tunneling limit, while increasing transmission leads to a strongly non‐sinusoidal current‐phase relation.

## Appendix C Toward Logical Error Rates

In this appendix, we make explicit the approximate order‐of‐magnitude relation discussed qualitatively in the main text between the junction material figures of merit and the physical and logical error rates relevant to fault‐tolerant quantum computation. The relation may be organized as a sequence of successive estimates,

$$\text{material FoM}\;⟶\;Q\;⟶\;T_{1},T_{2}\;⟶\;p_{\mathrm{coh}}\;⟶\;p\;⟶\;P_{L},$$

which corresponds to the three panels of Figure C1: the dielectric TLS branch $\tan\delta \;\to \;T_{1}$ (Figure C1a), the quasiparticle branch $x_{\mathrm{qp}}\;\to \;T_{1}$ (Figure C1b), and the surface‐code suppression law $p\;\to \;P_{L}$ (Figure C1c). Each relation employed below is either a standard identity or a result reported in the cited primary literature. We emphasize at the outset that the estimates are intended to establish characteristic scales rather than precise values. Because junction TLS can be sparse and strongly coupled, a continuum loss tangent need not predict the loss of an individual device at a particular operating frequency. We therefore distinguish device‐level effective loss tangents, inferred from measured quality factors, from STM loss tangents describing ensemble‐averaged absorption. Section C.1 discusses this distinction, while Section C.6 treats strongly coupled junction TLS as a possible source of qubit dropouts.

### C.1 Material Figure of Merit to Quality Factor

As discussed in the main text, TLS inside the junction barrier couple strongly to the qubit due to the relatively large electric field between the electrodes. Thus, their presence often results in non‐functioning qubits with order‐of‐magnitude smaller $T_{1}$, leading to yield issues. In addition, their sparse numbers necessitate different approaches compared to continuum TLS. Accounting for this detrimental effect often involves counting their statistics over an ensemble of measured qubits. If the yield loss is too severe, the transmon‐based device cannot be used to perform effective quantum error correction. Simulations of quantum error correction codes with site defects can be performed using advanced analysis [324]. Here, we first use the participation‐based loss framework to relate an effective junction loss to the measured quality factor and to illustrate the resulting scaling of relaxation‐ and coherence‐limited error. An effective loss inferred from an individual device characterizes that device under its measurement conditions; it is not automatically a measurement of the ensemble‐averaged STM loss tangent. We discuss this distinction below and consider the effects of discrete, strongly coupled TLS on quantum error correction in Section C.6.

Dielectric loss in a superconducting qubit is a *participation‐weighted* sum over the lossy regions of the device [325],

(C1)
$$\frac{1}{Q_{i}}\;=\;\tan\delta_{\mathrm{eff}}\;=\;\sum_{n}p_{n}\;\tan\delta_{n}^{0},$$

where $p_{n}$ denotes the fraction of electric field energy stored in region n and its intrinsic loss tangent $\tan\delta_{n}^{0}$. For the junction barrier, the amorphous ${\mathrm{AlO}}_{x}$ tunnel oxide hosts a bath of two‐level systems. The corresponding participation ratio, $p_{\mathrm{JJ}}\approx C_{J}/C_{\Sigma }$, with $C_{J}$ as the junction capacitance and $C_{\Sigma }$ as total shunt capacitance, is generally on the order of $10^{-3}-10^{-2}$ in a typical transmon and is therefore comparable to, or larger than, the participation of the distributed surface and interface dielectrics. Retaining only the junction contribution to Equation (C1), one obtains

(C2)
$$Q_{\mathrm{TLS}}\;=\;\frac{1}{p_{\mathrm{JJ}}\;\tan\delta_{\mathrm{JJ},\mathrm{eff}}}.$$

The junction‐material‐limited quantity is therefore the product $p_{\mathrm{JJ}}\tan\delta_{\mathrm{JJ},\mathrm{eff}}$, not either factor by itself. Here, $\tan\delta_{\mathrm{JJ},\mathrm{eff}}$ denotes the effective junction loss tangent under the specified operating and measurement conditions. If all measured relaxation loss is attributed to the junction, $1/(p_{\mathrm{JJ}}Q_{1})$ provides an upper bound on its effective loss tangent, because other relaxation channels may also contribute. This device‐level bound should be distinguished from the STM loss tangent inferred from TLS spectroscopy. For comparison, Ref. [124] applies the same decomposition to Nb/Al–AlOx/Nb trilayer transmons and finds an effective junction quality factor $Q_{j}\approx 10^{5}$ ($\tan\delta_{\mathrm{JJ},\mathrm{eff}}\approx 10^{-5}$) and concludes that its devices are not junction‐limited. Active strategies for reducing $p_{\mathrm{JJ}}\tan\delta_{\mathrm{JJ},\mathrm{eff}}$ include decreasing the junction area and $C_{J}$, increasing the low‐loss shunt capacitance, and reducing the intrinsic barrier loss through improved amorphous, crystalline, nitride, or van der Waals barriers. The first two strategies reduce $p_{\mathrm{JJ}}$ and are continuations of the capacitive‐shunting principle underlying the transmon. Reducing the junction area also lowers the expected number of strongly coupled TLS [61].

Reaching $p_{\mathrm{JJ}}=10^{-4}$ in a conventional tunnel‐junction transmon would, however, be aggressive. For example, $C_{\Sigma }=100\;fF$ would require $C_{J}=0.01\;fF$. For a representative ${\mathrm{AlO}}_{x}$ specific capacitance of $50\;\mathrm{fF}\mu m^{2}$, this corresponds to a junction area of approximately $2\times 10^{-4}\;\mu m^{2}$, or a square only about 14nm on a side; the precise value is process dependent. Alternatively, increasing $C_{\Sigma }$ to achieve the same ratio strongly reduces the charging energy, and hence the transmon anharmonicity. Accordingly, Figure C1a uses $p_{\mathrm{JJ}}=10^{-3}-10^{-2}$ as representative design values, and $10^{-4}$ as a prospective low‐participation bound, illustrating the benefit that further junction and circuit engineering could provide.

A continuum loss tangent describes a junction only when many defects couple to the qubit, which is not the typical case for junction barriers. Low‐field loss tangents of order $10^{-3}$ are often quoted for amorphous ${\mathrm{AlO}}_{x}$ [30]. However, the effective loss tangent of the barrier oxide layer following Equation C2 is much lower, with an estimated upper bound of approximately $4\times 10^{-8}$ [326]. Exploring junction TLS is especially intriguing in merged‐element transmon (MET), where the junction capacitance accounts for most of the charging energy, so the junction's participation ratio is close to unity. Ref. [207] stated that the barrier loss tangent should be smaller than $5\times 10^{-7}$, based on their approximate participation ratio of $p_{\mathrm{JJ}}\approx 0.93$ for the electric‐field energy. Attributing all measured loss to the junction gives the upper bound $\tan\delta_{\mathrm{JJ},\mathrm{eff}}\leq 1/\left(p_{\mathrm{JJ}}Q_{1}\right)$. With the best device‐mean $Q_{1}\approx 2.2\times 10^{6}$, this yields $\tan\delta_{\mathrm{JJ}},\mathrm{eff}\leq 5\times 10^{-7}$, and even the median $Q_{1}\approx 1.1\times 10^{6}$ of Table 4 gives $\leq 1\times 10^{-6}$. More recent METs with quality factors of up to $3.3\times 10^{6}$ bound $\tan\delta_{\mathrm{JJ},\mathrm{eff}}$ below about $3\times 10^{-7}$ when using the same argument [327]. Figure C1a illustrates how the junction‐TLS‐limited relaxation time scales with $p_{\mathrm{JJ}}\tan\delta_{\mathrm{JJ},\mathrm{eff}}$ via Equation (C2). They hold only at frequencies away from strongly coupled defects, which we treat separately as dropouts (Section C.6).

Direct TLS spectroscopy counts the defects in a barrier over a frequency band, rather than inferring a loss from the $T_{1}$ of a single device at one frequency. From such a study, Ref. [61] inferred an STM (Standard Tunneling Model) loss tangent $\tan\delta_{p}\approx 1.8\times 10^{-4}$ for the oxide in the large‐area parasitic junctions connected in series with the small junctions of the measured Xmon devices. The estimate was obtained from an observed TLS density of approximately $250\;G{\mathrm{Hz}}^{-1}\mu m^{-3}$, assuming a representative TLS dipole moment of 0.4eÅ and a dielectric constant $\varepsilon_{r}\approx 10$. It is the ensemble‐averaged absorption of a uniform continuum of dipoles. The authors noted that this value is approximately one order of magnitude below commonly quoted loss tangents, likely because weakly coupled TLS that were not resolved in swap spectroscopy were omitted. Extending this method to flux‐tunable MET, they resolved barrier defects at a density of 2000–2300 ${\mathrm{GHz}}^{-1}\mu m^{-3}$ with coupling strengths of up to 24 MHz (dipole moments up to 0.7eÅ) [327], which is equivalent to an STM loss tangent of $\tan\delta \approx 1.2\times 10^{-3}$, close to the bulk dielectric value.

Notably, for the qubits in Ref. [327], the $Q_{1}$‐based bound ($3\times 10^{-7}$) and the STM value ($1.2\times 10^{-3}$) differ by more than three orders of magnitude because they measure fundamentally different effects. Even a micrometer‐scale junction hosts only a few strongly coupled defects per gigahertz, so a qubit tuned between them sees only their far wings, while one that lies close to a defect is effectively disabled. The continuum loss tangent is the average over these situations and describes an individual junction only when many defects couple to the qubit. Continuum loss‐tangent values therefore cannot be transferred uncritically between bulk films, junction barriers, and device quality factors, and the sparse, strongly coupled junction TLS are better accounted for as a source of qubit dropouts (Section C.6).

### C.2 The Quasiparticle Branch

Nonequilibrium quasiparticles that tunnel across the junction constitute a distinct dissipation channel. The associated relaxation rate is given by the Catelani–Glazman expression [321, 328],

(C3)
$$\frac{1}{T_{1}^{\mathrm{qp}}}\;=\;\Gamma_{\mathrm{qp}}\;\approx \;x_{\mathrm{qp}}\;\sqrt{\frac{2\;\Delta \;\omega_{01}}{\pi^{2}\hbar }},$$

where $x_{\mathrm{qp}}=n_{\mathrm{qp}}/n_{\mathrm{cp}}$ is the normalized quasiparticle density and Δ is the superconducting gap ($\Delta_{\mathrm{Al}}\approx 200\;\mu \mathrm{eV}$). Equation (C3) defines a quasiparticle quality factor analogous to $Q_{\mathrm{TLS}}$, namely $Q_{\mathrm{qp}}=\omega_{01}T_{1}^{\mathrm{qp}}\propto 1/x_{\mathrm{qp}}$. Figure C1(b) displays Equation (C3) evaluated at $f_{01}=5\;\mathrm{GHz}$; since $\Gamma_{\mathrm{qp}}\propto \sqrt{f_{01}}$, the dependence on transition frequency is weak, and a single representative curve is sufficient. The vertical lines indicate reported quasiparticle densities: the hot‐quasiparticle distribution inferred to limit $T_{1}$ to ≈50μs [321], and representative steady‐state values reported for transmons [322] and fluxonium qubits [307, 323]. Because $T_{1}^{\mathrm{qp}}\propto x_{\mathrm{qp}}^{-1}$, improvements in dielectric loss that raise $T_{1}$ into the range of several hundred microseconds render quasiparticle tunneling comparably limiting, which provides the quantitative motivation for gap engineering.

### C.3 Coherence to Gate Error

A coherence‐limited (depolarizing) estimate of the local gate error is given as

(C4)
$$\begin{array}{cc} & p_{\mathrm{coh}}\equiv 1-F_{\mathrm{avg}}\;\approx \;\frac{t_{g}}{6}\;\left(\frac{1}{T_{1}}+\frac{2}{T_{2}}\right)\;=\;\frac{t_{g}}{3}\;\left(\frac{1}{T_{1}}+\frac{1}{T_{\phi }}\right),\\ & \frac{1}{T_{2}}=\frac{1}{2T_{1}}+\frac{1}{T_{\phi }}, \end{array}$$

where $T_{\phi }$ denotes the pure‐dephasing time. Taking $T_{2}=T_{1}$, Equation (C4) reduces to $p_{\mathrm{coh}}\approx t_{g}/\left(2T_{1}\right)$. Two representative cases illustrate the origin of the commonly quoted target $T_{1}≳100\;\mu s$: a single‐qubit gate of duration $t_{g}=25\;\mathrm{ns}$ at $T_{1}=100\;\mu s$ yields $p_{\mathrm{coh}}\approx 1.25\times 10^{-4}$, whereas an operation of duration $t_{g}=100\;\mathrm{ns}$ at the same $T_{1}$ yields $p_{\mathrm{coh}}\approx 5\times 10^{-4}$. Mapping this average infidelity onto the phenomenological physical‐error probability p in a surface‐code model is only an order‐of‐magnitude comparison and depends on the circuit and noise model.

A separate coherence‐only estimate can be given for a two‐qubit gate of duration $t_{2q}$,

(C5)
$$p_{\mathrm{coh}}^{\left(2q\right)}\;\approx \;\frac{t_{2q}}{5}\sum_{i=1}^{2}\left(\frac{1}{T_{1,i}}+\frac{2}{T_{2,i}}\right).$$

For identical qubits with $T_{1,i}=T_{2,i}=100\;\mu s$ and $t_{2q}=100$ ns, the gate error due to coherence is $p_{\mathrm{coh}}^{\left(2q\right)}\approx 1.2\times 10^{-3}$.

### C.4 Physical Error Budget

A surface‐code cycle comprises several operations, and the error budget of the 72‐qubit processor (Table S4 of Ref. [91]) attributes the per‐cycle error to a sum of contributions from distinct components. The measured per‐operation error probabilities and their contributions to the inverse suppression factor $\Lambda^{-1}$ are summarized in Table C1.

**TABLE C1:** Experimental error probabilities and contributions to $\Lambda^{-1}$ for individual error components. [91]

Component	$p_{\mathrm{expt}}$	$\Lambda^{-1}$ share
CZ gate (excl. crosstalk, leakage)	$2.8\times 10^{-3}$	41%
CZ stray‐interaction crosstalk	$5.5\times 10^{-4}$	11%
CZ leakage	$2.0\times 10^{-4}$	5%
Data‐qubit idle (during meas./reset)	$9\times 10^{-3}$	20%
Readout	$8\times 10^{-3}$	11%
Reset	$1.5\times 10^{-3}$	2%
Single‐qubit gate	$6.2\times 10^{-4}$	9%
Leakage (heating)	$2.5\times 10^{-4}$	1%

The two‐qubit (CZ) gates and their associated crosstalk and leakage account for approximately 57% of the budget; however, the data‐qubit idle error (∼20%), the readout and reset errors (∼13% combined), and the single‐qubit gate error (∼9%) are all comparable. Therefore, the per‐cycle error cannot be attributed to the two‐qubit gate alone. We note that this budget table should be used as a diagnostic decomposition for illustrative purposes rather than as exact accounting for all performance metrics. The part of this budget governed by the material figures of merit discussed so far is the component limited by $T_{1}$. The right‐hand axes of Figure C1a,b represent the per‐operation coherence‐limited average infidelity $p_{\mathrm{coh}}$ for a representative 100 ns operation, calculated using Equation (C4) with $T_{2}=T_{1}$.

### C.5 Physical to Logical Errors

For physical error rates below the threshold, the surface‐code logical error per cycle is described by the phenomenological suppression law [329],

(C6)
$$P_{L}\;\approx \;A{\left(\frac{p}{p_{\mathrm{th}}}\right)}^{\;(d+1)/2},\;A\approx 0.03,$$

in which d is the code distance and $p_{\mathrm{th}}$ the threshold. The surface code threshold for an idealized gate‐error model is $p_{\mathrm{th}}\approx 1.1-1.4\%$ [330], whereas a full circuit‐level model yields $p_{\mathrm{th}}=0.57\%$ [329]; the shaded band in Figure C1c denotes the interval 0.57−1.1%. The exponent (d+1)/2 gives rise to the error‐suppression factor

(C7)
$$\Lambda \;\equiv \;\frac{P_{L}\left(d\right)}{P_{L}\left(d+2\right)}\;\approx \;\frac{p_{\mathrm{th}}}{p},$$

so that operation at approximately one order of magnitude below the threshold, $p\approx p_{\mathrm{th}}/10$, yields Λ∼10 per increment of two in code distance; the attainment of algorithmically relevant logical error rates requires Λ≫1 [329]. The marker in Figure C1c is located at $p=p_{\mathrm{th}}/\Lambda$ using the measured value Λ=2.14 and the distance‐seven logical error of 0.143% per cycle of the 105‐qubit processor [91].

### C.6 Qubit Dropouts

As strongly coupled TLS often cause qubit dropouts, it is perhaps more appropriate to treat them as a yield issue. The results therefore also apply to general yield problems beyond TLS‐related physics, provided the dropouts are confined to data qubits. Here, we assume a simple model where the yield at a site i is either 0 or 1, meaning that the qubit is considered either functional or dropout, overlooking cases where a junction TLS is present with mild effects such as lowering the $T_{1}$ at the site by less than an order of magnitude. While the logical error rates discussed at the beginning of the section follow a simple prediction, the analysis below involves more complex numerical simulation of the surface code performance.

To survey the logical failure versus the dropout fraction, we model a missing data qubit as a known erasure. Surviving data qubits receive independent depolarizing errors, and the X and Z sectors are decoded separately using minimum‐weight perfect matching. This model intentionally excludes syndrome‐qubit and coupler yield, measurement errors, repeated syndrome rounds, leakage, and spatially correlated defects. It therefore reports logical failure per ideal correction instance, rather than computing a full circuit‐level logical error per surface‐code cycle. We note that this mainly serves to illustrate the effect of yield on the logical errors. The excluded mechanisms would further increase logical error and lower the tolerable dropout fraction.

Here, we define the disabled qubit fraction $f_{\mathrm{TLS}}$ as the probability that an individual data‐qubit site is out of service because a strongly coupled TLS sits within the damaging frequency window of its transition, which is separate from the operation‐error probability $p_{\mathrm{op}}$. If damaging TLS are Poisson‐distributed in area and frequency, the number in the damaging window around one qubit's transition is Poisson with mean $\lambda =\rho_{\mathrm{TLS}}\times A_{\mathrm{eff}}\times \Delta f$, where $\rho_{\mathrm{TLS}}$ is the TLS density in ${\left(\mathrm{GHz}\cdot \mu m^{2}\right)}^{-1}$, $A_{\mathrm{eff}}$ is the effective junction area in $\mu m^{2}$, and Δf is the frequency window in GHz, then the dropout (disabled) site fraction is $f_{\mathrm{TLS}}=1-e^{-\lambda }\approx \lambda$. Our model then assumes the following:

- *Disabled qubits are treated as known erasures*. A disabled qubit could in principle stay in service with a decoder that knows its elevated error rate. Disabling it discards all information about its state, so the erasure treatment gives a conservative estimate of the logical cost relative to keeping the qubit in service for a known defect location [324]. In the matching graph a known erasure is a zero‐weight edge, which is equivalent to merging the stabilizers adjacent to the disabled site into superstabilizers, as in the loss‐tolerant construction of Ref. [324].
- *Code‐capacity benchmark simulation*. Stabilizer measurements are ideal, and only data qubits are disabled. The idealized erasure model tolerates disabled fractions up to fifty percent [324]. Circuit‐level treatments, in which the stabilizers surrounding a defect are merged into superstabilizers, are far less tolerant: with 8% qubit fabrication errors the tolerable computational error rate falls to about 0.1% [331], and a 90% functional‐qubit yield suffices only if poorly fabricated chips are discarded [332]. We therefore restrict the scan to $f_{\mathrm{TLS}}\leq 10\%$, the upper end of which lies at the limit of circuit‐level operability. Results near that end should be read as optimistic.
- *Code‐capacity threshold*. Because stabilizer measurements are ideal, the relevant threshold is that of independent X/Z matching under depolarizing noise, $p_{\mathrm{th}}^{\mathrm{cc}}\approx 15\%$, which follows from the per‐sector matching threshold of 10.3% [333] and Equation C10. This is more than an order of magnitude above the circuit‐level 0.57–1.1% of Section C.5. The simulated values $p_{\mathrm{op}}=10^{-3}$, $3\times 10^{-3}$, and $10^{-2}$ therefore correspond to Λ≈150−200, 50−70, and 15−18, respectively, and $P_{L}$ in Figure C2 should be compared with Figure C1c at equal Λ, not at equal p.
- *Failure probability*. $P_{L}$ is a failure probability per ideal correction instance, rather than circuit‐level logical error per cycle.

We use unrotated planar surface codes of odd distance d, with data qubits on the sites (x,y), 0≤x,y≤2d−2,x≡y(mod2), so that $n_{d}=d^{2}+{\left(d-1\right)}^{2}$ (13, 41, and 85 for d = 3, 5, and 7). Z‐type and X‐type stabilizers occupy the odd–even and even–odd sites, respectively, and act on their nearest‐neighbor data qubits.

In the direct‐sampling cross‐check described below, each data qubit at site i is erased with probability $f_{\mathrm{TLS}}$ according to

(C8)
$$m_{i}\left(f_{\mathrm{TLS}}\right)=1\left[u_{i}<f_{\mathrm{TLS}}\right],$$

where $u_{i}\in \left[0,1\right)$ is a uniform random number associated with this site, and $m_{i}$ is Bernoulli distributed with $\mathrm{Pr}\left(m_{i}=1\right)=f_{\mathrm{TLS}}$. Conditioned on the number k of erased sites, this places them uniformly at random; the stratified estimator used for the reported results (below) draws such uniform k‐site sets directly. For each correction sample, an erased qubit is assigned a uniformly random Pauli from {I,X,Y,Z}. Equivalently, its X and Z components are independent fair bits. Its location is supplied to the decoder. This is the standard known‐erasure abstraction, where the error on the site is maximally uncertain but its location is known.

Each surviving data qubit independently undergoes the depolarizing channel

(C9)
$$E_{p_{\mathrm{op}}}\left(\rho \right)=\left(1-p_{\mathrm{op}}\right)\rho +\frac{p_{\mathrm{op}}}{3}\left(X\rho X+Y\rho Y+Z\rho Z\right).$$

Thus I is sampled with probability $1-p_{\mathrm{op}}$, and X,Y,Z are each sampled with probability $p_{\mathrm{op}}/3$. The marginal error probability in either matching sector is

(C10)
$$q_{sec}=\mathrm{Pr}\left(e_{X}=1\right)=\mathrm{Pr}\left(e_{Z}=1\right)=\frac{2p_{\mathrm{op}}}{3}.$$

The X and Z sectors are decoded separately with minimum‐weight perfect matching using PyMatching 2.3.1 [334]. For a surviving data‐qubit edge, the matching weight is the log likelihood ratio

(C11)
$$w_{\mathrm{surv}}=\log\left(\frac{1-q_{sec}}{q_{sec}}\right)=\log\left(\frac{1-2p_{\mathrm{op}}/3}{2p_{\mathrm{op}}/3}\right).$$

For a known erased edge, $q_{\sec}=1/2$, and hence

(C12)
$$w_{\mathrm{erase}}=\log\left(\frac{1/2}{1/2}\right)=0.$$

The zero weight tells the matching decoder that traversing a known erased site carries no likelihood penalty. Because every surviving edge carries the same weight $w_{\mathrm{surv}}$, the matching depends only on which sites are erased and not on $p_{\mathrm{op}}$. For every sample, PyMatching returns predicted logical parities ${\hat{\Lambda }}_{X}$ and ${\hat{\Lambda }}_{Z}$, which are compared with the actual parities $\Lambda_{X}=\left(\ell_{X}\cdot e_{X}\right)\;\mathrm{mod}\;2$ and $\Lambda_{Z}=\left(\ell_{Z}\cdot e_{Z}\right)\;\mathrm{mod}\;2$. Here $e_{X}$ and $e_{Z}$ are the binary X and Z components of the sampled error, and $\ell_{X}$ ($\ell_{Z}$) marks the data qubits on the left (top) boundary, so that it counts crossings of left‐to‐right X (top‐to‐bottom Z) logical strings. Logical failure is defined as

(C13)
$$F=1\;\left[\left({\hat{\Lambda }}_{X}\neq \Lambda_{X}\right)\;\vee \;\left({\hat{\Lambda }}_{Z}\neq \Lambda_{Z}\right)\right].$$

No defect map is discarded or post‐selected. Maps containing a damaging erasure pattern therefore contribute directly to the estimated failure rate.

Direct sampling of snapshots and errors resolves $P_{L}$ only down to roughly one failure per number of samples. At d=7 and $p_{\mathrm{op}}=10^{-3}$ the failure probability lies below $10^{-7}$ for $f_{\mathrm{TLS}}\leq 3\%$, so a direct simulation with $4\times 10^{6}$ samples per point leaves that region unresolved. The estimator used for the reported results instead stratifies by the number k of disabled sites and the number m of operation errors:

(C14)
PL(fTLS,pop)=∑k=0KB(k;nd,fTLS)∑m=0MB(m;nd−k,pop)Fk,m,B(j;n,r)=njrj(1−r)n−j,

where $F_{k,m}$ is the probability of logical failure when k disabled sites and m erroneous operable qubits are placed uniformly at random, each error carrying a uniformly random X, Y, or Z. Conditioned on k and m, this is exactly the distribution of the model above, so Equation (C14) is an identity up to the truncation at K and M. $F_{k,m}$ vanishes identically when m is below the correction radius of the code, reduced where disabled sites shorten the logical strings, so the smallest values of $P_{L}$ are determined by a few strata with moderate $F_{k,m}$ rather than by rare events.

For each code distance and each 1≤k≤K, we sample S=400 random disabled‐site configurations. For each configuration and each m, we generate $4\times 10^{5}/S$ error samples, giving a total of $4\times 10^{5}$ samples per (k,m) stratum. For k=0, there is only one disabled‐site configuration, and we generate $1.6\times 10^{6}$ error samples for each m to estimate the defect‐free baseline. The cutoffs K and M are the smallest values for which the binomial mass excluded at the largest $f_{\mathrm{TLS}}$ and $p_{\mathrm{op}}$ is below $10^{-12}$, giving (K,M)= (12, 7), (22,10), and (33,13) for d= 3, 5, and 7. The plotted uncertainty bands are nominal 95% bootstrap intervals obtained from 1000 replicates. Within each k stratum, disabled‐site configurations are resampled with replacement, and failure counts are resampled from binomial distributions using the corresponding empirical failure probabilities.

Under this procedure, strata with no observed failures contribute zero to both the point estimate and the bootstrap variation. A zero observed count, however, does not establish that the underlying failure probability is exactly zero, unless this follows from the error‐correction properties of the code. The bootstrap bands therefore do not account for possible unobserved failures in such strata and may underestimate uncertainty, particularly at the lowest logical failure probabilities. They should not be interpreted as conservative confidence bounds.

The estimator reproduces an exact enumeration of all $4^{13}$ Pauli patterns of the defect‐free d=3 code to within 0.2%, and agrees with direct sampling ($10^{3}$ snapshots × $4\times 10^{3}$ samples per point) at the 77 points where the latter records at least 30 failures (median ratio 1.03, largest deviation 2.9 standard errors).

As shown in Figure C2, at fixed code distance and operation fidelity, increasing qubit disability rates also increases the logical failure rates, even if all defect locations are known to the decoder. Meanwhile, we observe that larger code distance suppresses failure in the sampled range, as expected. Notably, at $p_{\mathrm{op}}=10^{-3}$, a 5% dropout fraction raises the d=7 failure probability from $6.9\times 10^{-10}$ to $3.0\times 10^{-7}$, comparable to a defect‐free d=5 code ($1.4\times 10^{-7}$). In this model, 5% dropouts therefore cost roughly one step (Δd=2) in code distance.

Finally, we provide an estimate of the dropout rate $f_{\mathrm{TLS}}$ of junctions fabricated using a standard process with nominal geometry. Following the standard tunneling model, a defect behaves as a small electric dipole that couples to the voltage across the junction. When its transition frequency lies close to the qubit frequency, the qubit loses energy to it and $T_{1}$ drops. We count the qubit as disabled when $T_{1}$ falls at least tenfold below its value $T_{1,0}$ without the defect.^1^ A defect therefore disables its qubit only if it lies within a certain frequency window around the qubit frequency. λ is the expected number of barrier defects inside that window. The width of the window follows from the relaxation rate of a qubit next to a single defect, $1/T_{1}=1/T_{1,0}+2g^{2}\Gamma /\left(\Gamma^{2}+\delta^{2}\right)$ [61], where g is the qubit–defect coupling, δ their detuning, and Γ the defect's linewidth. Requiring the second term to exceed $9/T_{1,0}$ gives a window of half‐width |δ|≈Wg, with $W={\left(2\Gamma T_{1,0}/9\right)}^{1/2}$. For a linewidth $\Gamma^{-1}\approx 100$ ns [61] and $T_{1,0}=100\;\mu s$, W≈15. The effective damaging window depends on the TLS lineshape, particularly its far‐detuned tails. For the illustrative estimate below, we adopt W=7 as a phenomenological modeling assumption, rather than the value W≈15 obtained from the preceding Lorentzian estimate. The resulting dropout probability is therefore conditional on this choice. A defect coupled with g/2π=10 MHz thus disables its qubit anywhere within about ±70 MHz. The window grows with the coupling, so the most strongly coupled defects matter most, and it grows as $T_{1,0}^{1/2}$, because the same absolute loss is a larger relative loss for a better qubit.

The standard tunneling model assumes that the defects are spread uniformly and couplings range from very weak up to a maximum $g_{\max}$ set by the largest dipole moment and the zero‐point electric field in the barrier [335]. Summing the windows of all defects gives the form $\lambda =\rho_{\mathrm{TLS}}A_{\mathrm{eff}}\Delta f$ introduced above, with $A_{\mathrm{eff}}$ the junction area and an effective window

(C15)
$$\Delta f\approx \frac{\pi }{4}W\frac{2g_{\max}}{2\pi }.$$

where the factor π/4 accounts for the spread of couplings and $\rho_{\mathrm{TLS}}$ is the defect density [335].

For the illustrative ${\mathrm{Al}/\mathrm{AlO}}_{x}$ junction estimate below, we adopt $\rho_{\mathrm{TLS}}=1\;{\left(\mathrm{GHz}\cdot \mu m^{2}\right)}^{-1}$ as a representative model input, rather than a universal material constant. Comparisons with spectroscopically measured TLS densities require consistent definitions of junction area and coupling threshold, so the adopted density should be treated as an assumption. For comparison, TLS density in phase‐qubit junctions was reported in Ref. [335] to be approximately ${\left(0.5\;\mathrm{GHz}\cdot \mu m^{2}\right)}^{-1}$. Meanwhile, for the SQUID MET studied in Ref. [327], the density is approximately $6.2\;{\left(\mathrm{GHz}\cdot \mu m^{2}\right)}^{-1}$, normalized to the total junction area. The zero‐point field in a transmon junction, $\approx 2.3\;\mathrm{kV}\;m^{-1}$ [142], and dipole moments of 0.4–1.3 eÅ [61, 335] give $g_{\max}/2\pi \approx 20-70\;\mathrm{MHz}$. Here we use a value of 40 MHz, as expected from Ref. [61]. Using W=7 and $g_{\max}/2\pi =40\;\mathrm{MHz}$, Equation (C15) gives an effective damaging frequency window Δf≈0.44GHz. With the adopted density $\rho_{\mathrm{TLS}}=1\;{\left(\mathrm{GHz}\cdot \mu m^{2}\right)}^{-1}$, the expected number of damaging TLS is $\lambda =\rho_{\mathrm{TLS}}A_{\mathrm{eff}}\Delta f$, and the disabled‐qubit fraction is $f_{\mathrm{TLS}}=1-\exp\left(-\lambda \right)$. For the baseline relaxation time $T_{1,0}=100\;\mu s$ considered here, these assumptions give $f_{\mathrm{TLS}}\approx 0.44\%$ for $A_{\mathrm{eff}}=0.01\;\mu m^{2}$.

For illustration, we consider junction areas of 0.02–$0.15\;\mu m^{2}$, representative of the Al/${\mathrm{AlO}}_{x}$/Al transmon junctions discussed in Refs. [61, 142]. Applying the model above to this area range gives $f_{\mathrm{TLS}}\approx 0.9$–6.4%. These calculated probabilities are conditional on the adopted model parameters; they are not experimentally measured dropout rates from the cited studies.

The estimates depend on the assumed TLS density, coupling distribution, lineshape, and criterion for disabling a qubit, and should be interpreted as illustrative, order‐of‐magnitude estimates rather than universal dropout rates for conventional transmons. Figure C2 illustrates how increasing the disabled‐qubit fraction degrades logical performance within the idealized code‐capacity model used here. A quantitative conversion of these material‐based estimates into an equivalent code‐distance cost requires evaluation at the corresponding dropout fractions and operation‐error probabilities.
